# Characterization of Genomic Alterations in Colorectal Liver Metastasis and Their Prognostic Value

**DOI:** 10.3389/fcell.2021.760618

**Published:** 2022-07-04

**Authors:** Xuanwen Bao, Kun Wang, Ming Liu, Bin Li, Hongwei Wang, Kemin Jin, Xiaoluan Yan, Hangyu Zhang, Quan Bao, Da Xu, Lijun Wang, Wei Liu, Yanyan Wang, Juan Li, Lijuan Liu, Weijia Fang, Baocai Xing

**Affiliations:** ^1^ Department of Medical Oncology, The First Affiliated Hospital, Zhejiang University School of Medicine and Key Laboratory of Cancer Prevention and Intervention, Ministry of Education, Hangzhou, China; ^2^ Key Laboratory of Carcinogenesis and Translational Research (Ministry of Education/Beijing), Hepatopancreatobiliary Surgery Department I, Peking University Cancer Hospital and Institute, Beijing, China

**Keywords:** colorectal liver metastases, genomic landscape, prognostics, overall survival, mutation signature

## Abstract

Colorectal liver metastases (CRLMs) are clinically heterogeneous lesions with poor prognosis. Genetic alterations play a crucial role in their progression. The traditional Fong clinical risk score (Fong-CRS) is commonly used for risk stratification and prognosis prediction. By identifying the genomic alterations of CRLMs, we aimed to develop a mutation-based gene-signature-based clinical score (mut-CS) system to improve clinical prognostication. Tumour tissues from 144 patients with CRLMs were analysed with next-generation sequencing (NGS). A mut-CS scoring system considering the unique mutation-based gene signature, primary site, and Fong-CRS was developed and could identify CRLM patients with poor prognosis. The mean time-dependent receiver operating characteristic curve AUC value of the mut-CS system was greater than that of previously established scoring measures (the Fong-CRS, the e-clinical score, the presence of concomitant RAS and TP53 mutations, and other clinical traits). Taking together, we identified a mutant signature that exhibits a strong prognostic effect for CRLMs. Traditional clinical scoring system characteristics were incorporated into the new mut-CS scoring system to help determine the appropriate treatment for CRLMs.

## Introduction

Colorectal cancer (CRC) is one of the most common cancers worldwide (Siegel et al., 2020). Metastases are the main cause of mortality in CRC patients (Ansa et al., 2018). Approximately 22% of CRCs are metastatic at initial diagnosis, and approximately 70% of patients will eventually develop metastatic relapse (Ansa et al., 2018). The liver is the most common location for distant metastasis of colorectal cancer due to its close proximity to the colorectum, with less frequent metastasis to the lung, bone, and nervous system (Manfredi et al., 2006; Engstrand et al., 2018; Wang et al., 2020). Vermaat et al. showed a significant difference in mutations in genes known to be involved in cancer pathways between primary CRC and matched liver metastases. They found significant genetic differences, with numerous losses and gains across the genome (Vermaat et al., 2012). Furthermore, colorectal liver metastases (CRLMs) are clinically heterogeneous lesions with poor prognosis (Manfredi et al., 2006; Turtoi et al., 2014). Hepatic resection improves the 5-years survival up to 50% (Abdalla et al., 2004; Rees et al., 2008). However, the varied prognoses and responses to therapy in CRLM require the stratification of patients according to risk for appropriate individualized treatments. Several clinical scores, which are commonly based on the preoperative carcinoembryonic antigen (CEA) value, resection margin, primary tumour site, tumour TNM stage (T: the size of the origin and whether it has invaded nearby tissue; N: nearby lymph nodes that are involved; M: distant metastasis), lymph node metastasis, and tumour size, are designed to evaluate the status and predict the prognosis of patients with CRLM (Nordlinger et al., 1996; Fong et al., 1999; Kattan et al., 2008). Among these, the Fong clinical risk score (Fong-CRS) is one of the most famous scoring systems (Fong et al., 1999). Some recent studies also raised several other scoring systems for better clinical decisions for CRLM patients. Kim et al. developed a scoring system based on synchronosity, CA19-9 level, number of liver metastases, largest size of liver metastases, resection margin of hepatic lesions, neutrophil-to-lymphocyte ratio, and prognostic nutritional index. They demonstrated that the novel scoring system would improve the sensitivity for the prediction of disease recurrence in CRLM patients (Kim et al., 2020). Liu et al. identified five independent risk factors for CRLM patients: tumour larger than 5 cm, more than one tumour, RAS mutation, primary lymph node metastasis, and primary tumour located on the right side (Liu et al., 2021). With the five identified risk factors, they constructed a nomogram to predict the progression-free survival of CRLM patients. Chen et al. established a modified tumour burden score (mTBS) by a mathematical equation with parameters including CRLM size, CRLM number, and unilobar or bilobar metastasis, which showed superior discriminatory capacity for stratifying CRLM patients with poor prognosis (Chen et al., 2020a). Nevertheless, most of the previous established scoring systems were constructed with the clinicopathological features of patients with CRLM. Considering the tumor heterogeneity and genomic difference between different patients, integrating genomic information may improve the predicting efficiency and provide new insight into the understanding the link between genome alteration, tumor progression and prognosis of CRLM patients. Next-generation sequencing (NGS) allows a comprehensive depiction of the genomic alterations (Chen et al., 2021; Wang et al., 2021), which helps determine molecular pathway alterations involved in CRLM and their relevance to prognosis. Recently, one study assessed the effect of 720 genes on the oncological outcome after resection of CRLMs and revealed that alterations of the SMAD family, as well as the RAS/RAF pathway, affected the prognosis of CRLM (Lang et al., 2019). A novel extended clinical score (e-CS) including RAS/RAF pathway and SMAD family alterations was developed to predict the overall survival of CRLM patients (Lang et al., 2019), which showed improved prediction efficiency compared with the traditional Fong-CRS system.

In this study, through the application of NGS, we identified genomic alterations in CRLM. Furthermore, associations between the primary sites of CRLM and gene mutations were identified in CRLM patients. To improve the prediction efficiency of mutation-based scoring system, we developed a novel mutation-based gene-signature-based clinical score (mut-CS).

With several machine learning concepts (e.g., regression, training and testing), we confirmed the robustness of the mut-CS system, which also showed better predicting efficiency than several previous established scoring systems. This study may provide helpful therapeutic information and improve the stratification of patients with CRLM.

## Materials and Methods

### Sample Collection

This study was approved by the Ethics Committee of Peking University Cancer Hospital and Institute in compliance with the ethical guidelines of the 1975 Declaration of Helsinki. The collection period is 8 years and all participants provided written informed consent to take part in the study. A total number of 146 patients were included and the mean age is 57.62 tumors were originated from left side of colon and 84 tumors were originated from right side of colon. The detailed patient information is provided in Supplementary Table S1.

### Next-Generation Sequencing

NGS analyses were performed in a centralized clinical testing centre according to protocols reviewed and approved by the Ethics Committee of Beijing Cancer Hospital. DNA was extracted from FFPE tumour tissues using a DNA Extraction Kit (QIAamp DNA FFPE Tissue Kit) according to the manufacturer’s protocols. Then, the DNA was sheared into 150–200 bp fragments with a BioruptorRPico Instrument (Diagenode, Seraing, Belgium). For each sample, 200–500 ng of FFPE DNA was then used for library preparation and quantification. Fragmented DNA libraries were constructed by the KAPA Hyper Prep Kit (KAPA Biosystems, Wilmington, Massachusetts, United States) following the manufacturer’s instructions. The final library was more than 600 ng, and fragment lengths were within the range of 250–400 base pairs (bp). DNA libraries were captured with a designed panel of 620 key cancer-related genes (GloriousMed, Shanghai, China). The list of 620 genes are shown in Supplementary Table S2. The captured samples were subjected to Illumina HiSeq X-Ten sequencing with a minimum depth of 500 ×.

### Mutation Calls

Illumina bcl2fastq (v2.19) software was used to demultiplex the sequencing data, followed by analysis with Trimmomatic (Bolger et al., 2014) to remove low-quality (quality<15) or N bases. Then, alignment of the data to the hg19 reference human genome was performed with the Burrows-Wheeler Aligner (bwa-mem) (Li, 2013), followed by processing using the Picard suite (available at https://broadinstitute.github.io/picard/) and the Genome Analysis Toolkit (GATK) with the default parameters (DePristo et al., 2011). Here, the Picard software can deduplicate the sequence. GATK-HaplotypeCaller was used to call germline SNPs, and GATK-Mutect2 was used to call somatic SNVs and indels. The Variant Allele Frequency (VAF) cut-off was set as 0.5%. Then, authenticity was confirmed in IGV (IGV Win 2.4.16). Known germline alternations in dbSNP were filtered out by comparison to the patient’s whole blood controls from each patient. Visualization of the genomic alterations was performed with maftools (Mayakonda et al., 2018). VEP software was used to annotate the mutation information, including the c-point and p-point information of the mutation under the HGVS rule, and the effect of the mutation on the protein function was also provided. ANNOVAR was further used to enrich the annotation results of mutations, including annotating the region where the mutation site is located, the type of SNV or Indel mutation in the exon region, the allele frequency of the mutation base at the mutation site in 1,000 Genomes, the mutation ID in dbSNP and so on. The information for annotated variants is in Supplementary Table S3. TP53- and KRAS-driven mutations were identified through the IntOGen-mutations platform (http://www.intogen.org/mutations/), which analysed 4,623 exomes from 13 cancer sites.

### Mutation-Based Gene Signature Construction

The least absolute shrinkage and selection operator (LASSO) algorithm was applied to establish the mutation-based gene signature. The L1-norm was applied to penalize the weight of the coefficients (Tibshirani, 1996). The cv.glmnet function from the glmnet package was used to build the model with the default parameters. Ten cross-validations were performed when constructing the scoring system. The key features were then selected while other features were penalized. The coefficient of the model was then identified with the coef function. A mut-CS formula was established by including individual mutation status (mutation: 1; wild-type: 0) weighted by the LASSO Cox coefficients and other clinical traits: size of metastases (1 point for >5 cm) + lymph node status (1 point for > positive) + primary site (1 point for right) + metastasis number (1 point for >1) + CEA value (1 point for >200) + metastasis status (1 point for synchronous) + $\;\underset{i}{\sum }coefficient\left(gene\;mutation_{i}\right)\left(from\;Figure\;4C\right)\times mutation\;status$ .

### Public Data Processing

NGS clinical data of the MSKCC cohort were downloaded from the Gene Expression Omnibus (GEO) database (http://www.ncbi.nlm.nih.gov/geo/).

### Statistics

Overall survival was calculated from the day of diagnosis or liver resection until the death of the patient. All statistical analyses were performed with R software and Python software. Survival analysis was performed with the “survival” package (Therneau and Lumley, 2014). The HR was determined with univariate or multivariate Cox regression analysis. The time-dependent receiver operating characteristic (tROC) curve quantifies the discriminative ability of a marker at each time point under consideration and was used to evaluate the AUC value during the follow-up period with the “survivalROC” package (Heagerty et al., 2013). Clinicopathological features with a significant effect in the univariate analysis were used to build a multivariate Cox model. *p* values < 0.05 were considered statistically significant.

## Results

### Genomic Alterations of Colorectal Liver Metastases

**FIGURE 1:**
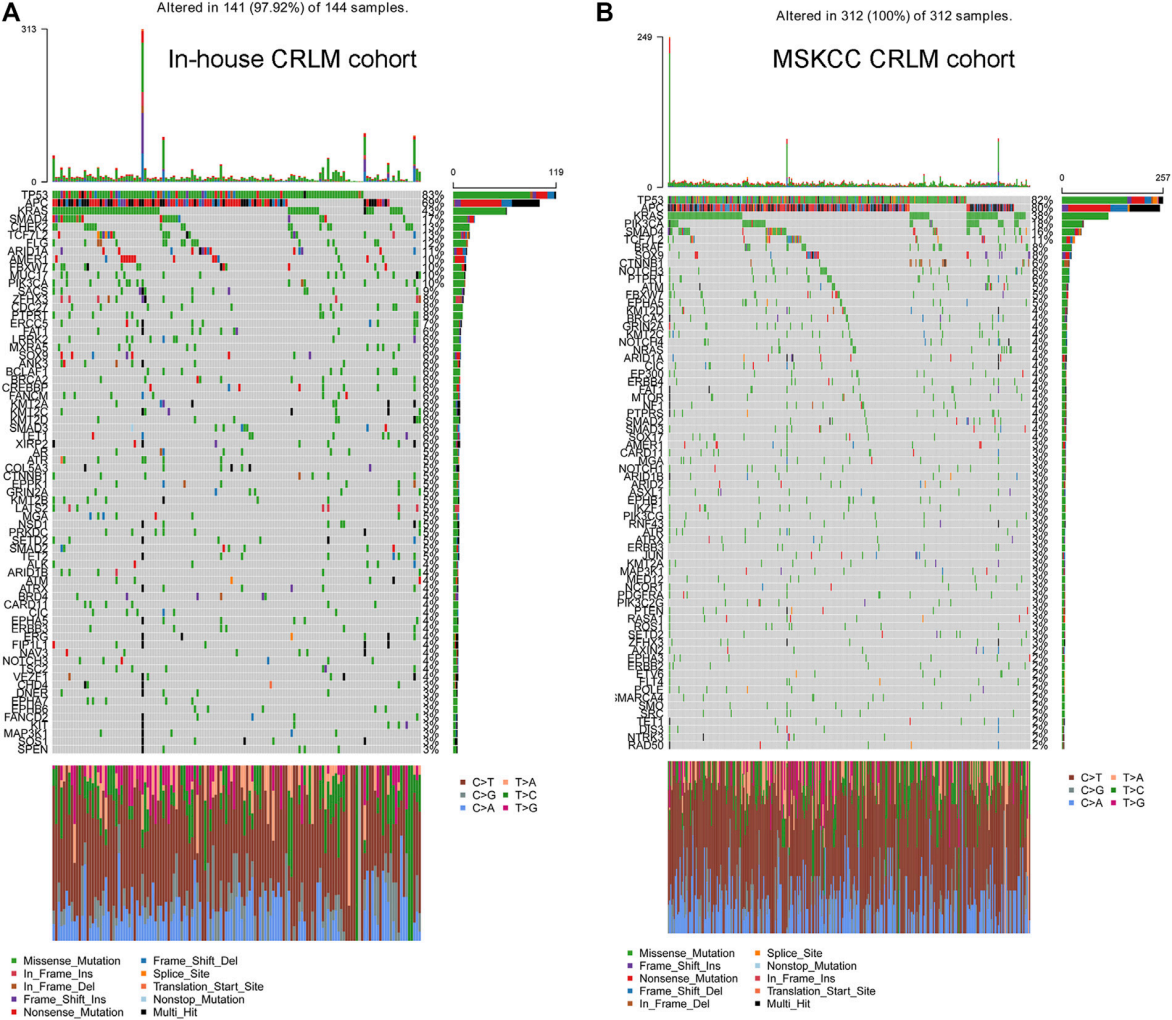
Distribution of commonly altered genes in the in-house CRLM **(A)** and SMKCC CRLM **(B)** cohorts and the frequencies of the mutations. CRLM: Colorectal liver metastases.

We analysed a total of 144 colorectal liver metastases. The detailed genomic alterations are shown in Figure 1A,B. *TP53* (83%), *APC* (69%), *KRAS* (43%) and *SMAD4* (17%) were the most frequent mutations in CRLMs. The major mutation type for *TP53* and *KRAS* was missense mutation, while the major mutation type of *APC* was frame-shift insertion. Furthermore, we extracted the data from patients with CRLMs from the MSKCC cohort (Yaeger et al., 2018) and reanalysed the genomic alterations of the samples. In the MSKCC cohort, *TP53* (82%), *APC* (80%), *KRAS* (38%) and *PIK3CA* (18%) were the genes most frequently mutated in patients with CRLMs (Supplementary Figure S1A,B).

Considering the synergistic contribution of RAS activation and the loss of p53 function in the malignant transformation of colorectal cancer cells, we also explored the effect of concomitant RAS and TP53 mutations on CRLM prognosis. In our results, the driver KRAS mutation was a predictive marker for overall survival in patients with CRLM (*p* = 0.007, HR = 1.87, 95% CI: 1.18–2.94 for OS from diagnosis of CRLM and *p* = 0.015, HR = 1.75, 95% CI: 1.11–2.76 for OS from hepatic resection) (Supplementary Figure S1A,B), while TP53 mutation showed an insignificant association with overall survival (*p* = 0.33, HR = 1.39, 95% CI: 0.71–2.72 for OS from diagnosis of CRLM; and *p* = 0.313, HR = 1.41, 95% CI: 0.72–2.75 for OS from hepatic resection) (Supplementary Figure S1C,D). Furthermore, dual mutations in RAS and TP53 were not an independent predictor of worse survival in CRLM patients (Figure S1E).

**FIGURE 2:**
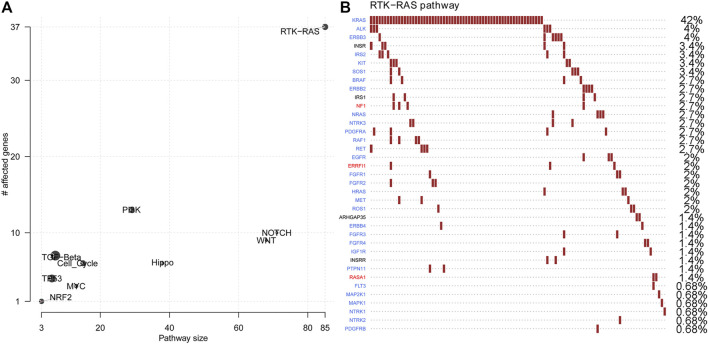
Oncogenic pathway changes and specific gene alterations in the RTK-RAS pathway **(A)** Oncogenic pathway changes in CRLMs **(B)** Specific gene alterations in the RTK-RAS pathway in CRLMs. CRLM: Colorectal liver metastases.

Next, oncogenic signalling pathways were also examined. RTK-RAS was the most affected oncogenic pathway and had the most gene mutations in the in-house CRLM cohort (Figure 2A, Supplementary Table S4). Of the genes in the RTK-RAS pathway with alterations, most were oncogenes in the CRLM cohort (Figure 2B). The patients with RAS-RAF pathway alterations had an unfavourable overall survival outcome compared with patients without RAS-RAF pathway alterations (*p* = 0.003, HR = 2.03, 95% CI: 1.27–3.24 for OS from diagnosis of CRLM; and *p* = 0.005, HR = 1.97, 95% CI: 1.23–3.15 for OS from hepatic resection) (Supplementary Figure S2A,B). We also analysed SMAD family gene mutations and the combined effect of SMAD pathway and RAS-RAF pathway alterations in our in-house cohort (Supplementary Table S4). Nevertheless, SMAD pathway alterations did not have a significant effect on the overall survival of patients with CRLM (Supplementary Figure S2C,D). Patients with alterations in both the SMAD pathway and the RTK-RAS pathway had a significantly different prognosis from patients without mutations in both pathways (pairwise comparison *p* = 0.03) (Supplementary Figure S2E).

### Genomic Difference by Primary Tumour Site

**FIGURE 3:**
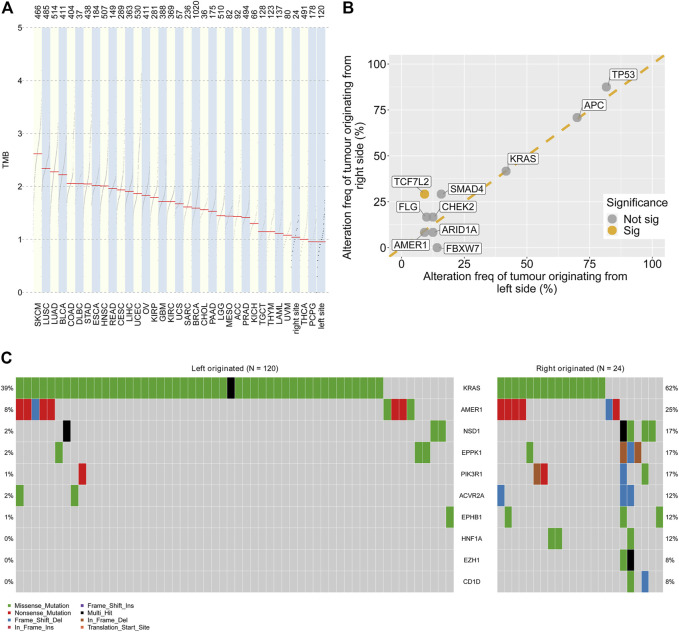
Genomic alterations by primary site **(A)** Comparison of the TMB values in CRLMs originating from the left and right sides of the colon with those in other tumour types. Data source: TCGA database **(B)** Genomic alteration frequency analysis by primary tumour site **(C)** Oncoplot of gene alterations with different frequencies in tumours originating from the left and right sides of the colon. TMB: tumour mutation burden; TCGA: The Cancer Genome Atlas.

The total gene mutation burden and the frequent mutation sites were analysed for left- and right-side colorectal liver metastases. The TMB values of CRLM and tumours from the TCGA cohort were compared. Greater tumour mutational burden (TMB) values were found in colorectal liver metastases originating from the right side than in those originating from the right side (Figure 3A). Of the 10 most significantly mutated genes in CRLM, *TCF7L2* showed a significantly higher alteration frequency in right side (29.2%) than in left side (9.2%) tumours (*p* = 0.013) (Figure 3B). *FBXW7* mutation frequency was marginally significantly different between CRLMs originating from the left (14.2%) and CRLMs originating from the right (0%) side (*p* = 0.07) (Figure 3B). Then, we compared the 50 most significantly mutated genes in CRLMs originating from the left and right sides. A total of 75 genes were included (Supplementary Table S5). Among the 75 genes, *KRAS, AMER1, NSD1, EPPK1, PIK3R1, ACVR2A, EPHB1, HNF1A, EZH1* and *CD1D* mutations were enriched in CRLMs originating from the right side (*p* value from chi-square tests) (Figure 3C, Supplementary Table S6).

### Establishment of a Mutation-Based Gene Signature for Overall Survival in CRLM Patients

**FIGURE 4:**
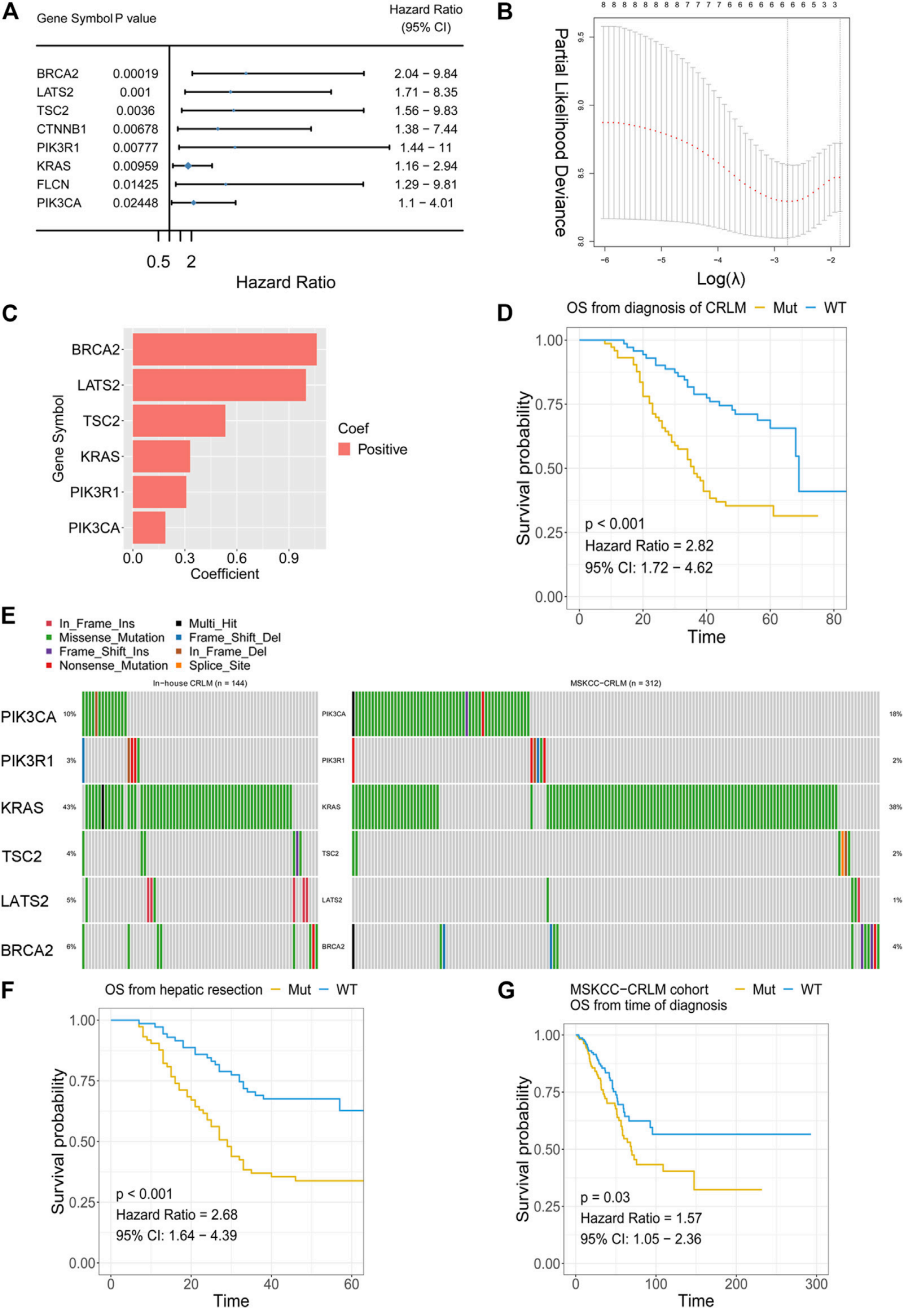
Mutation-based gene-signature construction **(A)** Selection of prognostic gene alterations **(B)** LASSO Cox regression feature selection **(C)** Distribution of the coefficients of the established gene signature **(D)** Patients with at least one gene mutation exhibited worse OS from the time of diagnosis of CRLM than those with no gene mutations in the in-house CRLM cohort **(E)** Oncoplot showing mutation types in the in-house CRLM cohort **(F)** Patients with at least one gene mutation exhibited worse OS after hepatic resection than those with no gene mutations in the in-house CRLM cohort **(G)** The external MSKCC-CRLM cohort validated the efficacy of the mutation-based gene signature. LASSO: least absolute shrinkage and selection operator; OS: overall survival; CRLM: colorectal liver metastases.

**FIGURE 5:**
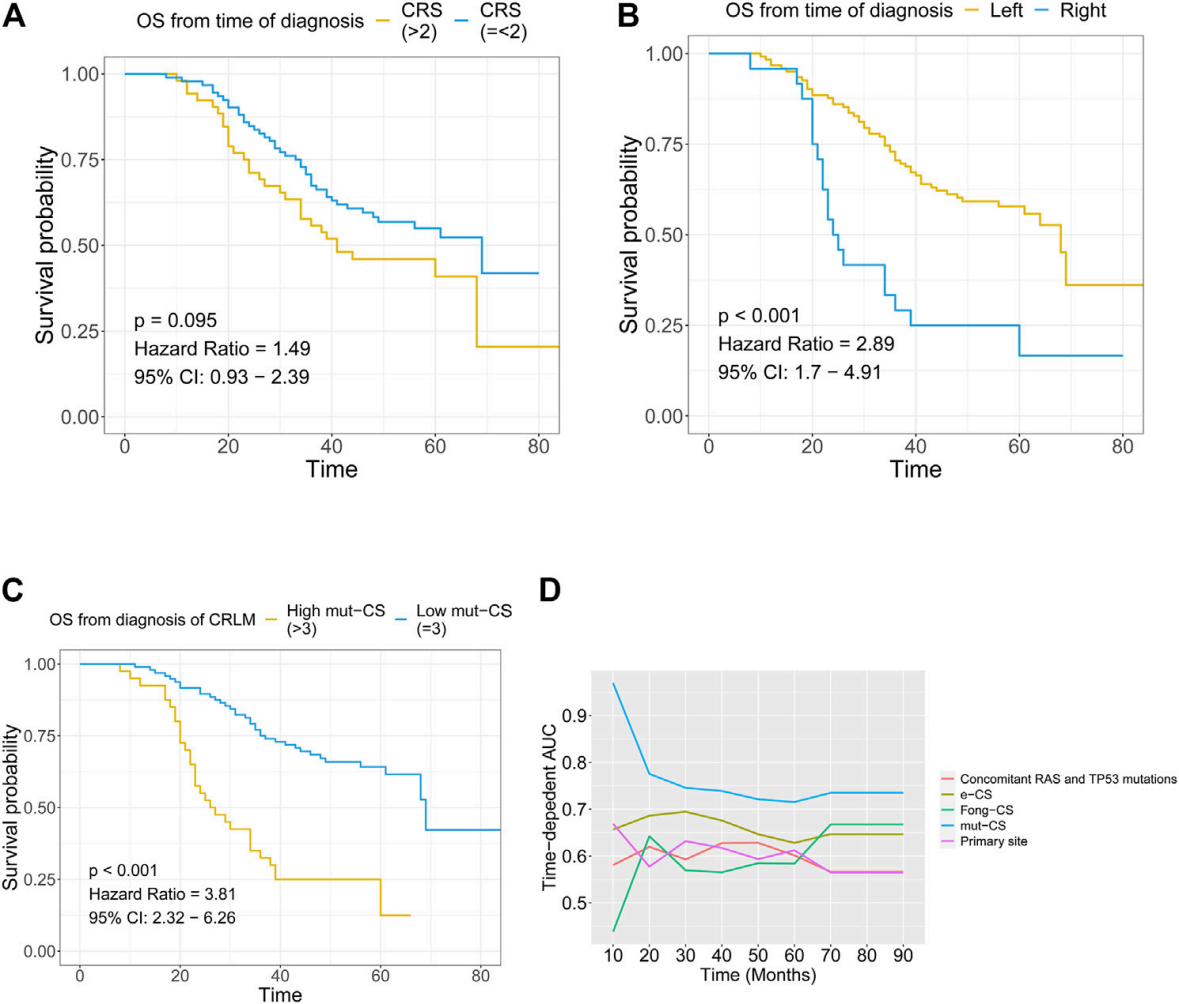
Combination of the mutation-based gene signature and clinical variables to establish the mut-CS scoring system to improve risk assessment and stratification **(A)** The association between Fong-CRS and OS from the time of diagnosis of CRLM **(B)** The association between the primary site of CRLM and OS from the time of diagnosis of CRLM **(C)** The association between mut-CS and OS from the time of diagnosis of CRLM **(D)** Time-dependent ROC analysis was performed to evaluate the predictive power of the mut-CS scoring system for OS in CRLM patients. OS: overall survival, CRLM: colorectal liver metastases.

The association between gene mutations and overall survival was analysed with univariate Cox analysis. The genes mutated in more than 3% of samples in the whole in-house cohort were filtered and then subjected to univariate Cox regression. In total, eight gene mutations showed unfavourable overall survival outcomes in patients with CRLM (Figure 4A). LASSO Cox regression was performed, and a mutation-based gene signature was built with six genes (Figures 4B,C). The patients from the in-house CRLM cohort were stratified into high-risk (at least one gene mutation) and low-risk groups (none of the six genes mutated). Kaplan–Meier plots and univariate Cox regression revealed that patients without the six gene mutations had favourable overall survival outcomes compared with patients with at least one gene mutation (*p* < 0.001, HR = 2.82) (Figure 4D). The detailed mutation types of the six genes are shown in Figure 5E. Missense mutation was the most frequent mutation type in *BRCA2, KRAS, TSC2 and PIK3CA*, while nonsense mutation was the most frequent in *PIK3R1* and *LATS2*. With the mutation-based gene signature, the patients could be stratified into high- and low-risk groups regarding the overall survival from hepatic resection in our in-house colorectal liver metastasis cohort (*p* < 0.001, HR = 2.68) (Figure 4F). To further validate the mutation-based gene signature, we applied it to an external cohort. The patients classified as high risk (at least one gene mutation) in the MSKCC colorectal liver metastasis cohort had unfavourable overall survival outcomes compared with the patients classified as low risk (no mutation) (*p* = 0.03, HR = 1.57) (Figure 4G).

### Combination of the Mutation-Based Gene Signature and Clinical Variables to Establish a Mut-CS Scoring System to Improve Risk Assessment and Stratification

Kaplan–Meier plots and univariate Cox regression revealed that Fong-CRS and primary site stratified patient survival in our in-house CRLM cohort (Figures 5A,B). In our in-house CRLM cohort, patients with left primary tumour sites had favourable survival outcomes compared with patients with right primary tumour sites (*p* < 0.001, HR = 2.89). In addition, patients with a higher Fong-CRS (>2) had marginally unfavourable survival compared with patients with a lower Fong-CRS (≤2) (*p* = 0.095, HR = 1.49).

The Fong-CRS is a useful tool in clinical practice. Recently, a study presented the concept of an extended clinical score (e-CS) considering the size of metastases, lymph node status and alterations in the RAS/RAF pathway, as well as the SMAD family (Lang et al., 2019). We established a novel mut-CS scoring system: size of metastases (1 point for >5 cm) + lymph node status (1 point for > positive) + primary site (1 point for right) + metastasis number (1 point for >1) + CEA value (1 point for >200) + metastasis status (1 point for synchronous) + $\;\underset{i}{\sum }coefficient\left(gene\;mutation_{i}\right)\left(from\;Figure\;4C\right)\times mutation\;status$ and compared the predictive power of the Fong-CRS score, the e-CS, primary site, concomitant *RAS* and *TP53* mutations and the mut-CS scoring system (Figure 5C). The average predictive power of the mut-CS system was greater than that of the Fong-CRS, e-CS, concomitant *RAS* and *TP53* mutations and other clinical traits (mean time-dependent ROC value of mut-CS = 0.75, cut off-value for mut-CS is 1.7, 1.1, 1.1, 1.1, and 1.0 for 1–5 years, respectively). Kaplan–Meier and univariate Cox regression analyses revealed that patients with a high mut-CS (>3) had poor prognosis (*p* < 0.01, HR = 3.81) (Figure 5D).

## Discussion

With the application of liver surgery and the resection of liver metastases, the majority of tumours can be removed (R0 resection). Traditionally, the criteria for patient selection and operation are clinically relevant, such as the Fong-CRS score, the response to preoperative chemotherapy and CEA levels and changes following chemotherapy. Among such prognostic factors, the Fong-CRS has been one of the most famous scoring systems since it was proposed in 1999. The Fong-CRS is constructed by several clinicopathological features, which are easily measured and assessed during clinical practice, which made the Fong-score as one of the commonly used tools. Due to the development of NGS, some studies have focused on improving the stratification and predictive capacity of the Fong-CRS system. Nevertheless, the Fong-CRS is still the top tool for determining risk and CRLM patient status. Brudvik et al. refined the Fong-CRS by including the lymph node status of the primary tumour, the size of the largest metastasis and the RAS mutation status (Brudvik et al., 2019). An e-CS that included size of metastases (1 point for >5 cm), lymph node status (1 point for lymph node positivity) and alterations in the RTK-RAS pathway (1 point for alteration), as well as the SMAD family (1 point for alteration), was raised by Lang et al. and exhibited accurate stratification capacity in patients with CRLMs (Lang et al., 2019). In this study, we identified a mutation-based gene signature including six gene mutations. Mutation-based gene-signature-based mut-CS stratified patients with CRLMs into high- and low-risk groups. The mut-CS system was developed based on the mutation-based gene signature identified in this study and the Fong-CRS. It is a new simple way to help clinicians make clinical decisions. With a calculation method similar to that of the traditional Fong-CRS (the sum of risk points for mutation status and clinical traits), each patient with CRLMs can obtain a risk score for prognosis prediction, which can help them receive individualized treatment. We also compared mut-CS with existing stratification methods based on concomitant *RAS* and *TP53* mutations, e-CS and the traditional CRS scoring system. The time-dependent ROC analysis suggested that mut-CS had higher predictive efficacy than e-CS, concomitant *RAS* and *TP53* mutations, primary site or Fong-CRS. Thus, mut-CS represents an improved version of the already established Fong-CRS system and may lead to further stratification of the treatment of CRLMs.

The mutation-based signature was constructed with *BRCA2, LATS2, TSC2, KRAS, PIK3R1* and *PIK3CA*. *BRCA2* is one of the crucial proteins involved in homologous recombination, which is the most accurate method of double-stranded DNA break repair. Germline mutations in *BRCA2* are highly penetrant for breast cancer and ovarian cancer (Antoniou et al., 2003), but the mechanism of *BRCA2* mutation in CRC is still not clear. One recent study shows *BRCA2* deletion triggers *TERRA* hyperexpression and alternative lengthening mechanisms (ALT) in colon cancer in presence of telomerase activity, which opens the question if patients bearing *BRCA2* mutation suitable for anti-telomerase therapies (Pompili et al., 2019). The continuous activation of Wnt signaling is one important mechanism for CRC initiation. *LATS2* suppresses oncogenic Wnt signaling by disrupting β-Catenin/BCL9 interaction (Li et al., 2013). *TSC2* inhibits cell growth by acting as a GTPase-activating protein toward Rheb, thereby inhibiting mTOR. The mutation in the *TSC2* tumor suppressor causes aberrant cell growth and therefore may contribute the tumor progression and metastasis of CRC (Inoki et al., 2006). *PIK3R1* which acting as the regulatory subunit of PI3K, stabilizes and inhibits the PI3K p110 catalytic subunit. PIK3R1 mutations are commonly occurred in CRC, therefore leading to CRC tumorgenesis (Philp et al., 2001). *PIK3CA* mutation is associated with poor prognosis among CRC patients and predicts response of colon cancer cells to the cetuximab (Jhawer et al., 2008; Ogino et al., 2009).

*TP53, APC, KRAS, SMAD, CHEK2* and *TCF7L2* were the most significantly altered genes in our CRLM cohort. The introduction of mutations in *APC, KRAS, PIK3CA, SMAD4* and *TP53*, four or five major driver genes for colorectal cancer, induces epithelial cell transformation, resulting in the development of transplantable tumours *in vivo* (Drost et al., 2015). The p53 mutation is found in approximately 60% of colorectal cancers, and a majority of mutations are missense-type at “hot spots”, suggesting an oncogenic role of mutant *p53* (Giannakis et al., 2016). *KRAS* mutation occurs early in CRC carcinogenesis and was observed in 27–43% of patients with CRC. *KRAS* mutation is considered a marker of resistance to EGFR inhibitor therapy (Lou et al., 2017). Among patients treated with cetuximab, the objective response rate for WT *KRAS* patients was 12.8%, compared with a 1.2% response rate for patients in the *KRAS* mutant group (Amado et al., 2008). Additional *KRAS* mutation activation induces more malignant phenotypes, such as EMT and metastasis (Bakir et al., 2020; Chen et al., 2020b). In our analysis, *KRAS* mutation was a prognostic indicator for overall survival, while *TP53* mutation showed no significant value for predicting overall survival in CRLM patients. One recent study also revealed that RAS mutation is a negative prognostic factor for OS, while *TP53* gene mutations do not seem to affect long-term outcomes, which is consistent with our results (Tsilimigras et al., 2018). Cooperation between mutated *TP53* and RAS activation plays a crucial role in the malignant progression of colorectal cancer cells (Parada et al., 1984; McMurray et al., 2008). The multiple steps of malignant transformation in colorectal cancer cells depend upon genes controlled synergistically by RAS activation and the loss of p53 function (McMurray et al., 2008). Recently, one study also confirmed that dual mutation of RAS/TP53 is an indicator of poor survival in patients with CRLMs (Chun et al., 2019). Thus, we analysed the association of overall survival and dual mutation of *TP53* and *KRAS* to evaluate the prognostic value of *TP53* and *KRAS* dual mutation. Nevertheless, the results showed that dual mutation of *KRAS* and *TP53* was not a predictive factor in our CRLM cohort. Furthermore, one study indicated that the most predictive parameters for poor survival were alterations in the SMAD family and RAS-RAF pathway in a colorectal liver metastasis cohort (Lang et al., 2019). In that study, combined consideration of alterations of the SMAD family, as well as the RTK-RAS pathway, could identify patients with poor survival (Lang et al., 2019). Nevertheless, we did not find that SMAD pathway alterations had a significant effect on the overall survival of patients with CRLM. This discrepant result may come from the criteria we used for patient selection, differences in chemotherapy response and/or ethnic differences, which warrants confirmation in larger patient cohorts.

The molecular differences between CRLMs originating from right-side and left-side primary colon cancer were evaluated. TMB values of CRLMSs from right-side were greater than that of CRLMs from left-side, which is in consistent with the previous studies (Kim et al., 2019; Bao et al., 2020). Of the 10 most commonly mutated genes in CRLMs, *TCF7L2* showed a significantly higher frequency in CRLMs originating from right-side colon cancer than in CRLMs originating from left-side colon cancer, while the *FBXW7* mutation frequency was marginally significantly different between tumours of these two origins. *TCF7L2* mutations included missense mutations (most frequent), splice site alterations, translation start site alterations, frame-shift insertions, nonsense mutations and frame-shift deletions in our in-house cohort. *TCF7L2* interacts with translocated β-catenin in the nucleus, thus leading to the conversion of *TCF7L2* into a transcription factor activator (Nelson and Nusse, 2004). The active form of *TCF7L2* induces the expression of c-MYC and other target genes, playing a crucial role in CRC carcinogenesis (Nelson and Nusse, 2004). However, the loss of TCF7L2 promotes migration and the invasion of human CRC by suppressing the expression of pro-oncogenic transcription factor RUNX2 and cell adhesion molecules, supporting tumour-suppressive functions (Wenzel et al., 2020). This paradox implies the complex roles of *TCF7L2* in CRC carcinogenesis. Alternative splicing creates *TCF7L2* isoforms with short or long C-terminal ends (Duval et al., 2000). The isoform with a long C-terminal end mediates transcriptional repression in CRC (Duval et al., 2000). Frameshift mutation leads to selective loss of TCF-4 isoforms with CtBP binding abilities (Cuilliere-Dartigues et al., 2006). One study revealed that, in MSI-H CRC that harbours a frameshift mutation of *TCF7L2*, CtBP is not able to repress *TCF7L2*-mediated transcription (Cuilliere-Dartigues et al., 2006). A higher frequency of *TCF7L2* mutations was found in CRLMs originating from the right side of the colon than in CRLMs originating from the left side of the colon. The higher frequency of *TCF7L2* alterations in CRLMs originating from the right side of the colon may also affect the WNT signalling pathway. *FBXW7* is a tumour repressor gene. *FBXW7* is the substrate recognition subunit for a ubiquitin ligase complex that can negatively regulate cell growth and metabolism by regulating the expression of c-Myc, cyclin E, Notch, TGIF and KLF5 (Welcker and Clurman, 2008). As *FBXW7* regulates a range of substrates, it may be a promising candidate for targeted therapeutics. A larger cohort may be required to explore the difference in *FBXW7* between CRLMs originating from the left and right sides of the colon as well as alteration-induced pathway changes. We also identified that the mutation burden was significantly higher in CRLMs originating from the right side of the colon than in CRLMs originating from the left side of the colon, which is consistent with data from primary colorectal cancer (Yaeger et al., 2018).

There are still some limitations to our study. In this study, we took blood as self-control. The existence of circulating tumour cells in blood may have reduced the reliability of our conclusion. Additionally, we proposed our scoring system of CRLM for clinical decision-making and validated it in an external cohort to further prove this system. However, the cohort size was still relatively small. Last, the number of CRLMs originating from the left site and right site of the colon was imbalanced.

In summary, we revealed the genomic alterations of CRLMs, which provided a potential biological explanation for clinical differences seen in metastases with different primary tumour sites and enabled the identification of prognostic factors. The mut-CS scoring system, which incorporates the Fong-CRS, primary site and mutation-based gene signature (*BRCA2, KRAS, TSC2, PIK3CA, PIK3R1* and *LATS2*), showed improved risk assessment for individual patients compared with the existing Fong-CRS. We hope that the mutation-based gene signature and mut-CS scoring system described in this report can be useful tools for identifying high-risk CRLM patients who may benefit from adjuvant therapies and contribute to the personalized management of CRLMs.

## Abbreviations

CRC, colorectal cancer; CRLMs, colorectal liver metastases; Fong-CRS, Fong clinical risk score; NGS, next-generation sequencing; e-CS, extended clinical score; mut-CS, mutation-based gene signature-based clinical score; GATK, Genome Analysis Toolkit; MAF, mutant allele frequency; LASSO, least absolute shrinkage and selection operator; GEO, Gene Expression Omnibus.

## Data Availability

The data presented in the study are deposited in the CNSA repository (https://db.cngb.org/cnsa/), accession number CNP0003154.

## Abbreviations

CRC, colorectal cancer; CRLMs, colorectal liver metastases; Fong-CRS, Fong clinical risk score; NGS, next-generation sequencing; e-CS, extended clinical score; mut-CS, mutation-based gene signature-based clinical score; GATK, Genome Analysis Toolkit; MAF, mutant allele frequency; LASSO, least absolute shrinkage and selection operator; GEO, Gene Expression Omnibus.

## References

[B1] Abdalla E. K. Vauthey J.-N. Ellis L. M. Ellis V. Pollock R. Broglio K. R. (2004). Recurrence and Outcomes Following Hepatic Resection, Radiofrequency Ablation, and Combined Resection/ablation for Colorectal Liver Metastases. Ann. Surg. 239 (6), 818–827. 10.1097/01.sla.0000128305.90650.71 15166961 PMC1356290

[B2] Amado R. G. Wolf M. Peeters M. Van Cutsem E. Siena S. Freeman D. J. (2008). Wild-type KRAS Is Required for Panitumumab Efficacy in Patients with Metastatic Colorectal Cancer. J. Clin. Oncol. 26 (10), 1626–1634. 18316791 10.1200/JCO.2007.14.7116

[B3] Ansa B. Coughlin S. Alema-Mensah E. Smith S. (2018). Evaluation of Colorectal Cancer Incidence Trends in the United States (2000-2014). Jcm 7 (2), 22. 10.3390/jcm7020022 PMC5852438 29385768

[B4] Antoniou A. Pharoah P. D. P. Narod S. Risch H. A. Eyfjord J. E. Hopper J. L. (2003). Average Risks of Breast and Ovarian Cancer Associated with BRCA1 or BRCA2 Mutations Detected in Case Series Unselected for Family History: a Combined Analysis of 22 Studies. Am. J. Hum. Genet. 72 (5), 1117–1130. 10.1086/375033 12677558 PMC1180265

[B5] Bakir B. Chiarella A. M. Pitarresi J. R. Rustgi A. K. (2020). EMT, MET, Plasticity, and Tumor Metastasis. Trends Cell Biology 30, 764. 10.1016/j.tcb.2020.07.003 PMC7647095 32800658

[B6] Bao X. Zhang H. Wu W. Cheng S. Dai X. Zhu X. (2020). Analysis of the Molecular Nature Associated with Microsatellite Status in colon Cancer Identifies Clinical Implications for Immunotherapy. J. Immunother. Cancer 8 (2), e001437. 10.1136/jitc-2020-001437 33028695 PMC7542666

[B7] Bolger A. M. Lohse M. Usadel B. (2014). Trimmomatic: a Flexible Trimmer for Illumina Sequence Data. Bioinformatics 30 (15), 2114–2120. 10.1093/bioinformatics/btu170 24695404 PMC4103590

[B8] Brudvik K. W. Jones R. P. Giuliante F. Shindoh J. Passot G. Chung M. H. (2019). RAS Mutation Clinical Risk Score to Predict Survival after Resection of Colorectal Liver Metastases. Ann. Surg. 269 (1), 120–126. 10.1097/sla.0000000000002319 28549012

[B9] Chen D. Bao X. Zhang R. Ding Y. Zhang M. Li B. (2021). Depiction of the Genomic and Genetic Landscape Identifies CCL5 as a Protective Factor in Colorectal Neuroendocrine Carcinoma. Br. J. Cancer 125, 1–9. 10.1038/s41416-021-01501-y 34331023 PMC8476633

[B10] Chen P. Li X. Zhang R. Liu S. Xiang Y. Zhang M. (2020). Combinative Treatment of β-elemene and Cetuximab Is Sensitive to KRAS Mutant Colorectal Cancer Cells by Inducing Ferroptosis and Inhibiting Epithelial-Mesenchymal Transformation. Theranostics 10 (11), 5107–5119. 10.7150/thno.44705 32308771 PMC7163451

[B11] Chen Y. Chang W. Ren L. Chen J. Tang W. Liu T. (2020). Comprehensive Evaluation of Relapse Risk (CERR) Score for Colorectal Liver Metastases: Development and Validation. The oncologist 25 (7), e1031–e1041. 10.1634/theoncologist.2019-0797 32181531 PMC7356794

[B12] Chun Y. S. Passot G. Yamashita S. Nusrat M. Katsonis P. Loree J. M. (2019). Deleterious Effect of RAS and Evolutionary High-Risk TP53 Double Mutation in Colorectal Liver Metastases. Ann. Surg. 269 (5), 917–923. 10.1097/sla.0000000000002450 28767562 PMC7462436

[B13] Cuilliere-Dartigues P. El-Bchiri J. Krimi A. Buhard O. Fontanges P. Fléjou J. F. (2006). TCF-4 Isoforms Absent in TCF-4 Mutated MSI-H Colorectal Cancer Cells Colocalize with Nuclear CtBP and Repress TCF-4-Mediated Transcription. Oncogene 25 (32), 4441–4448. 10.1038/sj.onc.1209471 16547505

[B14] DePristo M. A. Banks E. Poplin R. Garimella K. V. Maguire J. R. Hartl C. (2011). A Framework for Variation Discovery and Genotyping Using Next-Generation DNA Sequencing Data. Nat. Genet. 43 (5), 491–498. 10.1038/ng.806 21478889 PMC3083463

[B15] Drost J. Van Jaarsveld R. H. Ponsioen B. Zimberlin C. Van Boxtel R. Buijs A. (2015). Sequential Cancer Mutations in Cultured Human Intestinal Stem Cells. Nature 521 (7550), 43–47. 10.1038/nature14415 25924068

[B16] Duval A. Rolland S. Tubacher E. Bui H. Thomas G. Hamelin R. (2000). The Human T-Cell Transcription Factor-4 Gene: Structure, Extensive Characterization of Alternative Splicings, and Mutational Analysis in Colorectal Cancer Cell Lines. Cancer Res. 60 (14), 3872–3879. 10919662

[B17] Engstrand J. Nilsson H. Strömberg C. Jonas E. Freedman J. (2018). Colorectal Cancer Liver Metastases - a Population-Based Study on Incidence, Management and Survival. BMC cancer 18 (1), 78–11. 10.1186/s12885-017-3925-x 29334918 PMC5769309

[B18] Fong Y. Fortner J. Sun R. L. Brennan M. F. Blumgart L. H. (1999). Clinical Score for Predicting Recurrence after Hepatic Resection for Metastatic Colorectal Cancer. Ann. Surg. 230 (3), 309. 10.1097/00000658-199909000-00004 10493478 PMC1420876

[B19] Giannakis M. Mu X. J. Shukla S. A. Qian Z. R. Cohen O. Nishihara R. (2016). Genomic Correlates of Immune-Cell Infiltrates in Colorectal Carcinoma. Cel Rep. 15 (4), 857–865. 10.1016/j.celrep.2016.03.075 PMC4850357 27149842

[B20] Heagerty P. Saha P. P S. (2013). survivalROC: Time-dependent ROC Curve Estimation from Censored Survival Data R Package Version 1.0. San Francisco.

[B21] Inoki K. Ouyang H. Zhu T. Lindvall C. Wang Y. Zhang X. (2006). TSC2 Integrates Wnt and Energy Signals via a Coordinated Phosphorylation by AMPK and GSK3 to Regulate Cell Growth. Cell 126 (5), 955–968. 10.1016/j.cell.2006.06.055 16959574

[B22] Jhawer M. Goel S. Wilson A. J. Montagna C. Ling Y.-H. Byun D.-S. (2008). PIK3CA Mutation/PTEN Expression Status Predicts Response of colon Cancer Cells to the Epidermal Growth Factor Receptor Inhibitor Cetuximab. Cancer Res. 68 (6), 1953–1961. 10.1158/0008-5472.can-07-5659 18339877 PMC3972216

[B23] Kattan M. W. Gönen M. Jarnagin W. R. DeMatteo R. D'Angelica M. Weiser M. (2008). A Nomogram for Predicting Disease-specific Survival after Hepatic Resection for Metastatic Colorectal Cancer. Ann. Surg. 247 (2), 282–287. 10.1097/sla.0b013e31815ed67b 18216534

[B24] Kim J. E. Chun S.-M. Hong Y. S. Kim K.-p. Kim S. Y. Kim J. (2019). Mutation burden and I index for Detection of Microsatellite Instability in Colorectal Cancer by Targeted Next-Generation Sequencing. J. Mol. Diagn. 21 (2), 241–250. 10.1016/j.jmoldx.2018.09.005 30389464

[B25] Kim W.-J. Lim T.-W. Kang S.-H. Park P.-J. Choi S.-B. Lee S.-i. (2020). Development and Validation of Novel Scoring System for the Prediction of Disease Recurrence Following Resection of Colorectal Liver Metastasis. Asian J. Surg. 43 (2), 438–446. 10.1016/j.asjsur.2019.06.001 31439461

[B26] Lang H. Baumgart J. Heinrich S. Tripke V. Passalaqua M. Maderer A. (2019). Extended Molecular Profiling Improves Stratification and Prediction of Survival after Resection of Colorectal Liver Metastases. Ann. Surg. 270 (5), 799–805. 10.1097/sla.0000000000003527 31634180

[B27] Li H. (2013). Aligning Sequence Reads, Clone Sequences and Assembly Contigs with BWA-MEM. [Preprint]. Cambridge, MA.

[B28] Li J. Chen X. Ding X. Cheng Y. Zhao B. Lai Z.-c. (2013). LATS2 Suppresses Oncogenic Wnt Signaling by Disrupting β-Catenin/BCL9 Interaction. Cel Rep. 5 (6), 1650–1663. 10.1016/j.celrep.2013.11.037 PMC3897473 24360964

[B29] Liu W. Zhang W. Xu Y. Li Y-H. Xing B-C. (2021). A Prognostic Scoring System to Predict Survival Outcome of Resectable Colorectal Liver Metastases in This Modern Era. Ann. Surg. Oncol. 28, 1–10. 10.1245/s10434-021-10143-6 34023948

[B30] Lou E. D'Souza D. Nelson A. C. (2017). Therapeutic Response of Metastatic Colorectal Cancer Harboring aKRASMissense Mutation after Combination Chemotherapy with the EGFR Inhibitor Panitumumab. J. Natl. Compr. Canc Netw. 15 (4), 427–432. 10.6004/jnccn.2017.0043 28404754 PMC6295659

[B31] Manfredi S. Lepage C. m. Hatem C. Coatmeur O. Faivre J. Bouvier A.-M. (2006). Epidemiology and Management of Liver Metastases from Colorectal Cancer. Ann. Surg. 244 (2), 254–259. 10.1097/01.sla.0000217629.94941.cf 16858188 PMC1602156

[B32] Mayakonda A. Lin D.-C. Assenov Y. Plass C. Koeffler H. P. (2018). Maftools: Efficient and Comprehensive Analysis of Somatic Variants in Cancer. Genome Res. 28 (11), 1747–1756. 10.1101/gr.239244.118 30341162 PMC6211645

[B33] McMurray H. R. Sampson E. R. Compitello G. Kinsey C. Newman L. Smith B. (2008). Synergistic Response to Oncogenic Mutations Defines Gene Class Critical to Cancer Phenotype. Nature 453 (7198), 1112–1116. 10.1038/nature06973 18500333 PMC2613942

[B34] Nelson W. J. Nusse R. (2004). Convergence of Wnt, SS-Catenin, and Cadherin Pathways. Science 303 (5663), 1483–1487. 10.1126/science.1094291 15001769 PMC3372896

[B35] Nordlinger B. Guiguet M. Vaillant J.-C. Balladur P. Boudjema K. Bachellier P. (1996). Surgical Resection of Colorectal Carcinoma Metastases to the Liver: a Prognostic Scoring System to Improve Case Selection, Based on 1568 Patients. Cancer 77 (7), 1254–1262. 10.1002/(sici)1097-0142(19960401)77:7<1254:aid-cncr5>3.0.co;2-i 8608500

[B36] Ogino S. Nosho K. Kirkner G. J. Shima K. Irahara N. Kure S. (2009). PIK3CA Mutation Is Associated with Poor Prognosis Among Patients with Curatively Resected colon Cancer. Jco 27 (9), 1477–1484. 10.1200/jco.2008.18.6544 PMC2659340 19237633

[B37] Parada L. F. Land H. Weinberg R. A. Wolf D. Rotter V. (1984). Cooperation between Gene Encoding P53 Tumour Antigen and Ras in Cellular Transformation. Nature 312 (5995), 649–651. 10.1038/312649a0 6390217

[B38] Philp A. J. Campbell I. G. Leet C. Vincan E. Rockman S. P. Whitehead R. H. (2001). The Phosphatidylinositol 3'-kinase P85alpha Gene Is an Oncogene in Human Ovarian and colon Tumors. Cancer Res. 61 (20), 7426–7429. 11606375

[B39] Pompili L. Maresca C. Dello Stritto A. Biroccio A. Salvati E. (2019). BRCA2 Deletion Induces Alternative Lengthening of Telomeres in Telomerase Positive colon Cancer Cells. Genes 10 (9), 697. 10.3390/genes10090697 PMC6771010 31510074

[B40] Rees M. Tekkis P. P. Welsh F. K. S. O'Rourke T. John T. G. (2008). Evaluation of Long-Term Survival after Hepatic Resection for Metastatic Colorectal Cancer. Ann. Surg. 247 (1), 125–135. 10.1097/sla.0b013e31815aa2c2 18156932

[B41] Siegel R. L. Miller K. D. Goding Sauer A. Fedewa S. A. Butterly L. F. Anderson J. C. (2020). Colorectal Cancer Statistics. CA Cancer J Clin. 10.3322/caac.21601 32133645

[B42] Therneau T. M. Lumley T. (2014). Package ‘survival’. Survival Analysis Published on CRAN. [Preprint]. Rochester, MN, 3.

[B43] Tibshirani R. (1996). Regression Shrinkage and Selection via the Lasso. J. R. Stat. Soc. Ser. B (Methodological) 58 (1), 267–288. 10.1111/j.2517-6161.1996.tb02080.x

[B44] Tsilimigras D. I. Ntanasis-Stathopoulos I. Bagante F. Moris D. Cloyd J. Spartalis E. (2018). Clinical Significance and Prognostic Relevance of KRAS, BRAF, PI3K and TP53 Genetic Mutation Analysis for Resectable and Unresectable Colorectal Liver Metastases: A Systematic Review of the Current Evidence. Surg. Oncol. 27 (2), 280–288. 10.1016/j.suronc.2018.05.012 29937183

[B45] Turtoi A. Blomme A. Debois D. Somja J. Delvaux D. Patsos G. (2014). Organized Proteomic Heterogeneity in Colorectal Cancer Liver Metastases and Implications for Therapies. Hepatology 59 (3), 924–934. 10.1002/hep.26608 23832580

[B46] Vermaat J. S. Nijman I. J. Koudijs M. J. Gerritse F. L. Scherer S. J. Mokry M. (2012). Primary Colorectal Cancers and Their Subsequent Hepatic Metastases Are Genetically Different: Implications for Selection of Patients for Targeted Treatment. Clin. Cancer Res. 18 (3), 688–699. 10.1158/1078-0432.ccr-11-1965 22173549

[B47] Wang H. Ding Y. Chen Y. Jiang J. Chen Y. Lu J. (2021). A Novel Genomic Classification System of Gastric Cancer via Integrating Multidimensional Genomic Characteristics. Gastric Cancer 24, 1227–1241. 10.1007/s10120-021-01201-9 34095982 PMC8502137

[B48] Wang J. Li S. Liu Y. Zhang C. Li H. Lai B. (2020). Metastatic Patterns and Survival Outcomes in Patients with Stage IV colon Cancer: A Population‐based Analysis. Cancer Med. 9 (1), 361–373. 10.1002/cam4.2673 31693304 PMC6943094

[B49] Welcker M. Clurman B. E. (2008). FBW7 Ubiquitin Ligase: a Tumour Suppressor at the Crossroads of Cell Division, Growth and Differentiation. Nat. Rev. Cancer 8 (2), 83–93. 10.1038/nrc2290 18094723

[B50] Wenzel J. Rose K. Haghighi E. B. Lamprecht C. Rauen G. Freihen V. (2020). Loss of the Nuclear Wnt Pathway Effector TCF7L2 Promotes Migration and Invasion of Human Colorectal Cancer Cells. Oncogene 39 (19), 3893–3909. 10.1038/s41388-020-1259-7 32203164 PMC7203011

[B51] Yaeger R. Chatila W. K. Lipsyc M. D. Hechtman J. F. Cercek A. Sanchez-Vega F. (2018). Clinical Sequencing Defines the Genomic Landscape of Metastatic Colorectal Cancer. Cancer cell 33 (1), 125–136. 10.1016/j.ccell.2017.12.004 29316426 PMC5765991

