# Supplementary material for: Characterization of Genomic Alterations in Colorectal Liver Metastasis and Their Prognostic Value
Source: Front Cell Dev Biol. 2022 Jul 4;9:760618. doi: 10.3389/fcell.2021.760618 (PMC9289210; doi:10.3389/fcell.2021.760618)
Supplement: Supplementary file 2 [file Table1.DOCX]

Supplementary table S1: Clinical characteristics of 144 CRC patients with hepatic metastasis

|  | Number of patients，n (%) |
| --- | --- |
| Overall | 144 (100%) |
| Age  <65  ≥65 | 112 (78%)  32 (22%) |
| Gender  Male  Female | 92 (64%)  52 (36%) |
| Colorectal tumor sidedness  Left  Right | 120 (83%)  24 (17%) |
| Extent of metastatic tumor resection  R0  R1 | 113 (78%)  31 (22%) |
| Time to metastases  Synchronous  Metachronous | 91 (63%)  53 (37%) |
| Previous neoadjuvant chemotherapy  Yes  No | 116 (81%)  28 (19%) |
| Primary tumor  Node-negative  Node-positive  NA | 55 (38%)  81 (56%)  8 (6%) |
| Disease-free interval  <12 mo  ≥12 mo  NA | 121 (84%)  22 (15%)  1 (1%) |
| Intrahepatic disease  ≤5 cm  >5 cm | 132 (92%)  12 (8%) |
| Number of hepatic metastases  1  >1 | 50 (35%)  94 (65%) |
| Preoperative CEA level  ≤200 ng/ml  >200 ng/ml | 139 (97%)  5 (3%) |
| 1-year survival  Reach  Not reach | 139 (97%)  5 (3%) |
| 3-year survival  Reach  Not reach | 91 (63%)  53 (37%) |

Data are n (%). NA: not available.
