# Supplementary figures and images for: Characterization of Genomic Alterations in Colorectal Liver Metastasis and Their Prognostic Value
Source: Front Cell Dev Biol. 2022 Jul 4;9:760618. doi: 10.3389/fcell.2021.760618 (PMC9289210; doi:10.3389/fcell.2021.760618)

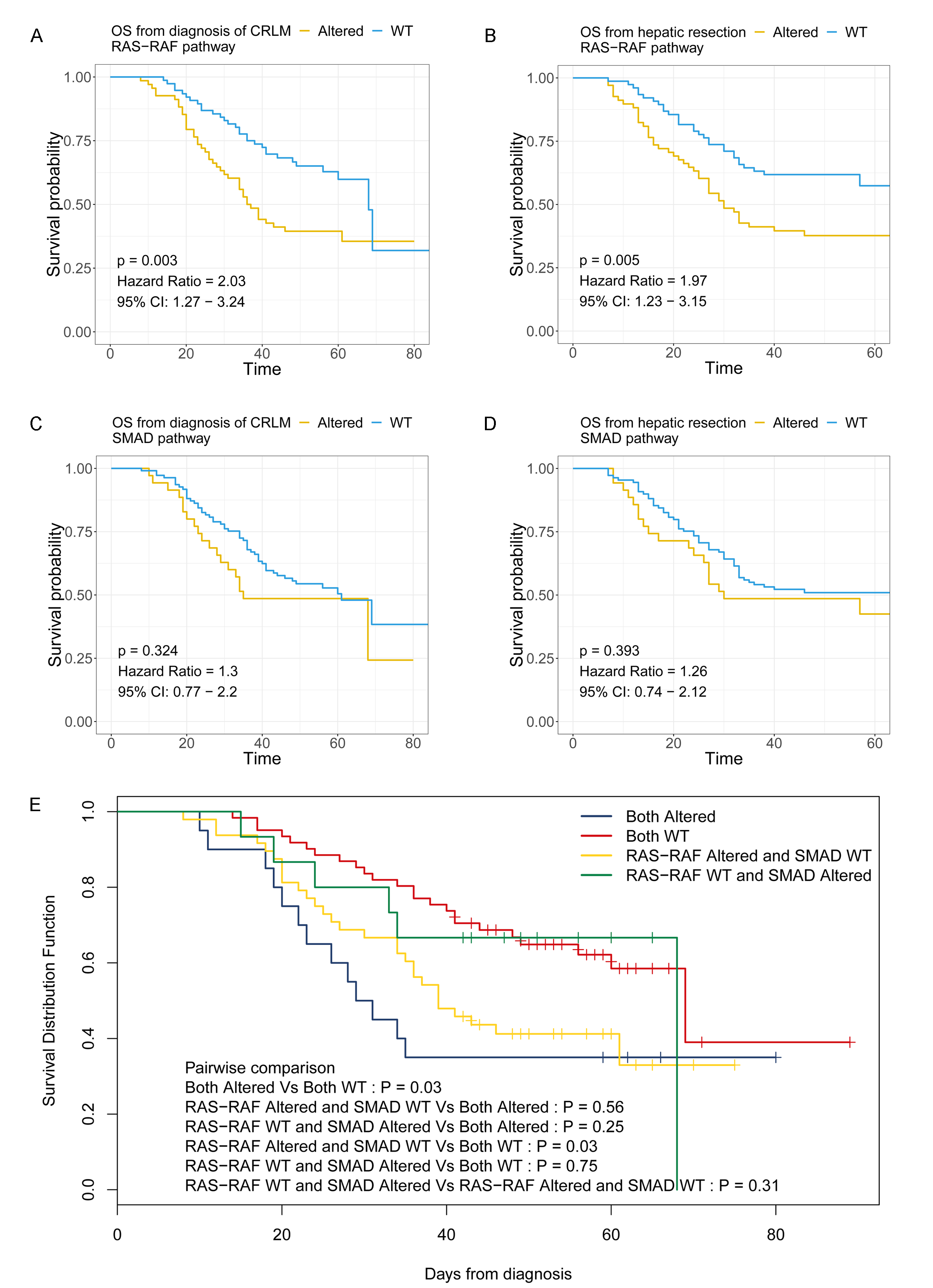

Supplement: Supplementary file 3 [file Image2.TIF]

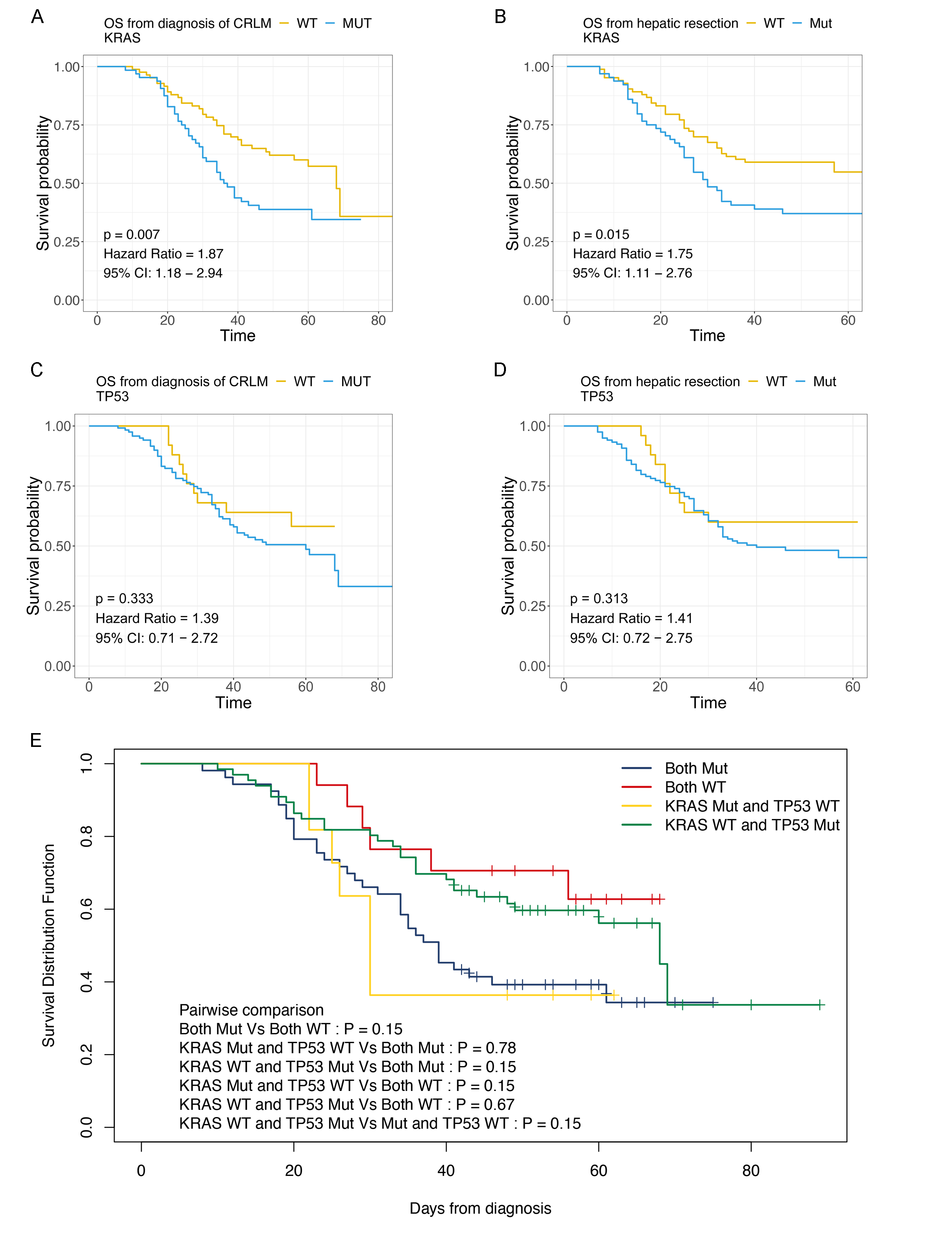

Supplement: Supplementary file 4 [file Image1.TIF]
