# Supplementary material for: Characterization of Genomic Alterations in Colorectal Liver Metastasis and Their Prognostic Value
Source: Front Cell Dev Biol. 2022 Jul 4;9:760618. doi: 10.3389/fcell.2021.760618 (PMC9289210; doi:10.3389/fcell.2021.760618)
Supplement: Supplementary file 5 [file Table5.DOCX]

Top50 most frequent mutations

|  | Left side origination | Right side origination |
| --- | --- | --- |
| 1 | TP53 | TP53 |
| 2 | APC | APC |
| 3 | KRAS | KRAS |
| 4 | SMAD4 | AMER1 |
| 5 | FLG | PIK3CA |
| 6 | FBXW7 | NSD1 |
| 7 | TCF7L2 | MUC17 |
| 8 | ARID1A | ZFHX3 |
| 9 | CHEK2 | CHEK2 |
| 10 | CDC27 | EPPK1 |
| 11 | MUC17 | PIK3R1 |
| 12 | SACS | SMAD4 |
| 13 | PIK3CA | TCF7L2 |
| 14 | AMER1 | SACS |
| 15 | PTPRT | ATRX |
| 16 | ERCC5 | CTNNB1 |
| 17 | TET1 | KMT2C |
| 18 | FANCM | ACVR2A |
| 19 | SOX9 | BRCA2 |
| 20 | ZFHX3 | EPHB1 |
| 21 | FAT1 | ERBB3 |
| 22 | ANK3 | HNF1A |
| 23 | SMAD3 | LRRK2 |
| 24 | FIP1L1 | MXRA5 |
| 25 | KMT2A | NAV3 |
| 26 | COL5A3 | SETD2 |
| 27 | XIRP2 | KMT2D |
| 28 | AR | VEZF1 |
| 29 | LRRK2 | CUX1 |
| 30 | BCLAF1 | FLG |
| 31 | CREBBP | KMT2A |
| 32 | KMT2B | XIRP2 |
| 33 | KMT2D | EP300 |
| 34 | LATS2 | EZH1 |
| 35 | MXRA5 | PTCH1 |
| 36 | SMAD2 | PTPRD |
| 37 | SOS1 | RAD54L |
| 38 | TET2 | ALK |
| 39 | FANCD2 | ATR |
| 40 | PRKDC | BCLAF1 |
| 41 | EPHA5 | CCNE1 |
| 42 | KMT2C | CD1D |
| 43 | ATM | CIC |
| 44 | BRCA2 | CNBD1 |
| 45 | EPHA7 | CNKSR1 |
| 46 | EPHB6 | CREBBP |
| 47 | GRIN2A | ERCC5 |
| 48 | TSC2 | ERG |
| 49 | ARID1B | FAT1 |
| 50 | ATR | GRIN2A |
