# Supplementary material for: Characterization of Genomic Alterations in Colorectal Liver Metastasis and Their Prognostic Value
Source: Front Cell Dev Biol. 2022 Jul 4;9:760618. doi: 10.3389/fcell.2021.760618 (PMC9289210; doi:10.3389/fcell.2021.760618)
Supplement: Supplementary file 6 [file Table3.DOCX]

| ID | summary | Percentage |
| --- | --- | --- |
| Frame_Shift_Del | 233 | 10.90% |
| Frame_Shift_Ins | 207 | 9.69% |
| In_Frame_Del | 58 | 2.71% |
| In_Frame_Ins | 60 | 2.81% |
| Missense_Mutation | 1309 | 61.25% |
| Nonsense_Mutation | 241 | 11.28% |
| Nonstop_Mutation | 1 | 0.05% |
| Splice_Site | 15 | 0.70% |
| Translation_Start_Site | 13 | 0.61% |
| total | 2137 | 100.00% |
