# Supplementary material for: Characterization of Genomic Alterations in Colorectal Liver Metastasis and Their Prognostic Value
Source: Front Cell Dev Biol. 2022 Jul 4;9:760618. doi: 10.3389/fcell.2021.760618 (PMC9289210; doi:10.3389/fcell.2021.760618)
Supplement: Supplementary file 7 [file Table4.DOCX]

| Pathway | Tumor_Sample_Barcode | Chromosome | Start Position | End Position | Reference Allele | Tumor Seq Allele2 | Hugo Symbol | Variant_Classification | AAChange.refGene | t_vaf |
| --- | --- | --- | --- | --- | --- | --- | --- | --- | --- | --- |
| RAS-RTK | 1810321 | 38287455 | 38287455 | C | T | FGFR1 | Missense_Mutation | FGFR1:NM_001174063:exon3:c.G103A:p.G35R,FGFR1:NM_001174065:exon3:c.G103A:p.G35R,FGFR1:NM_015850:exon3:c.G103A:p.G35R,FGFR1:NM_023110:exon3:c.G103A:p.G35R,FGFR1:NM_001174064:exon4:c.G79A:p.G27R,FGFR1:NM_001174067:exon4:c.G202A:p.G68R | 41.90% | SNP |
|  | 1810321 | 1801064 | 1801064 | G | A | FGFR3 | Missense_Mutation | FGFR3:NM_000142:exon3:c.G193A:p.G65R,FGFR3:NM_001163213:exon3:c.G193A:p.G65R,FGFR3:NM_022965:exon3:c.G193A:p.G65R | 39.90% | SNP |
|  | 1810381 | 25398284 | 25398284 | C | T | KRAS | Missense_Mutation | KRAS:NM_004985:exon2:c.G35A:p.G12D,KRAS:NM_033360:exon2:c.G35A:p.G12D | 39.30% | SNP |
|  | 1810382 | 149502735 | 149502735 | G | A | PDGFRB | Missense_Mutation | PDGFRB:NM_002609:exon15:c.C2053T:p.R685C | 68.50% | SNP |
|  | 1810382 | 115256530 | 115256530 | G | T | NRAS | Missense_Mutation | NRAS:NM_002524:exon3:c.C181A:p.Q61K | 37.40% | SNP |
|  | 1810383 | 25380275 | 25380275 | T | G | KRAS | Missense_Mutation | KRAS:NM_004985:exon3:c.A183C:p.Q61H,KRAS:NM_033360:exon3:c.A183C:p.Q61H | 33.00% | SNP |
|  | 1810385 | 176517963 | 176517963 | G | A | FGFR4 | Missense_Mutation | FGFR4:NM_022963:exon4:c.G461A:p.R154H,FGFR4:NM_001291980:exon5:c.G461A:p.R154H,FGFR4:NM_002011:exon5:c.G461A:p.R154H,FGFR4:NM_213647:exon5:c.G461A:p.R154H | 50.20% | SNP |
|  | 1810386 | 25398284 | 25398284 | C | A | KRAS | Missense_Mutation | KRAS:NM_004985:exon2:c.G35T:p.G12V,KRAS:NM_033360:exon2:c.G35T:p.G12V | 14.50% | SNP |
|  | 1810386 | 88799348 | 88799348 | A | T | NTRK3 | Missense_Mutation | NTRK3:NM_001320134:exon1:c.T37A:p.W13R,NTRK3:NM_001007156:exon3:c.T37A:p.W13R,NTRK3:NM_001012338:exon3:c.T37A:p.W13R,NTRK3:NM_001243101:exon3:c.T37A:p.W13R,NTRK3:NM_002530:exon3:c.T37A:p.W13R | 5.60% | SNP |
|  | 1810387 | 8075425 | 8075425 | G | A | ERRFI1 | Missense_Mutation | ERRFI1:NM_018948:exon3:c.C145T:p.P49S | 32.50% | SNP |
|  | 1810389 | 25398284 | 25398284 | C | T | KRAS | Missense_Mutation | KRAS:NM_004985:exon2:c.G35A:p.G12D,KRAS:NM_033360:exon2:c.G35A:p.G12D | 15.50% | SNP |
|  | 1810391 | 29416316 | 29416316 | G | T | ALK | Missense_Mutation | ALK:NM_004304:exon29:c.C4637A:p.A1546E | 10.10% | SNP |
|  | 1810391 | 55146617 | 55146617 | G | A | PDGFRA | Missense_Mutation | PDGFRA:NM_001347827:exon16:c.G2291A:p.R764H,PDGFRA:NM_001347829:exon16:c.G2291A:p.R764H,PDGFRA:NM_001347830:exon16:c.G2330A:p.R777H,PDGFRA:NM_006206:exon16:c.G2291A:p.R764H,PDGFRA:NM_001347828:exon17:c.G2366A:p.R789H | 9.60% | SNP |
|  | 1810391 | 25398284 | 25398284 | C | T | KRAS | Missense_Mutation | KRAS:NM_004985:exon2:c.G35A:p.G12D,KRAS:NM_033360:exon2:c.G35A:p.G12D | 16.70% | SNP |
|  | 1810396 | 55138642 | 55138642 | C | T | PDGFRA | Missense_Mutation | PDGFRA:NM_001347827:exon9:c.C1319T:p.T440M,PDGFRA:NM_001347829:exon9:c.C1319T:p.T440M,PDGFRA:NM_001347830:exon9:c.C1358T:p.T453M,PDGFRA:NM_006206:exon9:c.C1319T:p.T440M,PDGFRA:NM_001347828:exon10:c.C1394T:p.T465M | 41.90% | SNP |
|  | 1810723 | 25398281 | 25398281 | C | T | KRAS | Missense_Mutation | KRAS:NM_004985:exon2:c.G38A:p.G13D,KRAS:NM_033360:exon2:c.G38A:p.G13D | 6.70% | SNP |
|  | 1810724 | 25398279 | 25398279 | C | T | KRAS | Missense_Mutation | KRAS:NM_004985:exon2:c.G40A:p.V14I,KRAS:NM_033360:exon2:c.G40A:p.V14I | 72.70% | SNP |
|  | 1810724 | 227662763 | 227662763 | G | A | IRS1 | Missense_Mutation | IRS1:NM_005544:exon1:c.C692T:p.T231M | 45.30% | SNP |
|  | 1810727 | 25398281 | 25398281 | C | T | KRAS | Missense_Mutation | KRAS:NM_004985:exon2:c.G38A:p.G13D,KRAS:NM_033360:exon2:c.G38A:p.G13D | 15.60% | SNP |
|  | 1810730 | 115256530 | 115256530 | G | T | NRAS | Missense_Mutation | NRAS:NM_002524:exon3:c.C181A:p.Q61K | 10.20% | SNP |
|  | 1810731 | 25398281 | 25398281 | C | T | KRAS | Missense_Mutation | KRAS:NM_004985:exon2:c.G38A:p.G13D,KRAS:NM_033360:exon2:c.G38A:p.G13D | 16.50% | SNP |
|  | 1810732 | 25398281 | 25398281 | C | T | KRAS | Missense_Mutation | KRAS:NM_004985:exon2:c.G38A:p.G13D,KRAS:NM_033360:exon2:c.G38A:p.G13D | 44.60% | SNP |
|  | 1810734 | 25398284 | 25398284 | C | A | KRAS | Missense_Mutation | KRAS:NM_004985:exon2:c.G35T:p.G12V,KRAS:NM_033360:exon2:c.G35T:p.G12V | 20.60% | SNP |
|  | 1810736 | 47422638 | 47422638 | G | A | ARHGAP35 | Missense_Mutation | ARHGAP35:NM_004491:exon1:c.G706A:p.V236M | 6.30% | SNP |
|  | 1810737 | 117730774 | 117730774 | G | A | ROS1 | Missense_Mutation | ROS1:NM_002944:exon4:c.C260T:p.A87V | 26.20% | SNP |
|  | 1810737 | 25398281 | 25398281 | C | T | KRAS | Missense_Mutation | KRAS:NM_004985:exon2:c.G38A:p.G13D,KRAS:NM_033360:exon2:c.G38A:p.G13D | 28.90% | SNP |
|  | 1810741 | 38275768 | 38275768 | G | A | FGFR1 | Missense_Mutation | FGFR1:NM_001174066:exon9:c.C1141T:p.R381C,FGFR1:NM_023105:exon9:c.C1141T:p.R381C,FGFR1:NM_023106:exon9:c.C1135T:p.R379C,FGFR1:NM_001174063:exon10:c.C1402T:p.R468C,FGFR1:NM_001174065:exon10:c.C1402T:p.R468C,FGFR1:NM_015850:exon10:c.C1402T:p.R468C,FGFR1:NM_023110:exon10:c.C1408T:p.R470C,FGFR1:NM_001174064:exon11:c.C1378T:p.R460C,FGFR1:NM_001174067:exon11:c.C1501T:p.R501C | 46.30% | SNP |
|  | 1810741 | 87322806 | 87322806 | G | A | NTRK2 | Missense_Mutation | NTRK2:NM_001018064:exon4:c.G407A:p.R136H,NTRK2:NM_001007097:exon5:c.G407A:p.R136H,NTRK2:NM_001018065:exon5:c.G407A:p.R136H,NTRK2:NM_001018066:exon5:c.G407A:p.R136H,NTRK2:NM_001291937:exon7:c.G407A:p.R136H,NTRK2:NM_006180:exon7:c.G407A:p.R136H | 51.30% | SNP |
|  | 1810742 | 7117331 | 7117331 | G | C | INSR | Missense_Mutation | INSR:NM_001079817:exon21:c.C3849G:p.H1283Q,INSR:NM_000208:exon22:c.C3885G:p.H1295Q | 48.80% | SNP |
|  | 1810742 | 110435519 | 110435519 | C | - | IRS2 | Frame_Shift_Del | IRS2:NM_003749:exon1:c.2882delG:p.G961fs | 50.20% | DEL |
|  | 1810742 | 110435522 | 110435535 | GAGCCCAGCGACGA | - | IRS2 | Frame_Shift_Del | IRS2:NM_003749:exon1:c.2866_2879del:p.S956fs | 48.90% | DEL |
|  | 1810742 | 25398284 | 25398284 | C | T | KRAS | Missense_Mutation | KRAS:NM_004985:exon2:c.G35A:p.G12D,KRAS:NM_033360:exon2:c.G35A:p.G12D | 29.20% | SNP |
|  | 1810743 | 99454630 | 99454630 | T | A | IGF1R | Missense_Mutation | IGF1R:NM_000875:exon7:c.T1549A:p.Y517N,IGF1R:NM_001291858:exon7:c.T1549A:p.Y517N | 54.40% | SNP |
|  | 1810745 | 86682643 | 86682643 | G | T | RASA1 | Nonsense_Mutation | RASA1:NM_002890:exon23:c.G2848T:p.E950X,RASA1:NM_022650:exon23:c.G2317T:p.E773X | 57.90% | SNP |
|  | 1810745 | 28644725 | 28644725 | C | T | FLT3 | Missense_Mutation | FLT3:NM_004119:exon2:c.G68A:p.G23E | 27.30% | SNP |
|  | 1810748 | 25380275 | 25380275 | T | G | KRAS | Missense_Mutation | KRAS:NM_004985:exon3:c.A183C:p.Q61H,KRAS:NM_033360:exon3:c.A183C:p.Q61H | 2.30% | SNP |
|  | 1810749 | 56486844 | 56486844 | G | A | ERBB3 | Missense_Mutation | ERBB3:NM_001982:exon11:c.G1258A:p.G420S | 24.20% | SNP |
|  | 1810749 | 88726682 | 88726682 | C | A | NTRK3 | Missense_Mutation | NTRK3:NM_001320134:exon3:c.G362T:p.R121I,NTRK3:NM_001320135:exon3:c.G68T:p.R23I,NTRK3:NM_001007156:exon5:c.G362T:p.R121I,NTRK3:NM_001012338:exon5:c.G362T:p.R121I,NTRK3:NM_001243101:exon5:c.G362T:p.R121I,NTRK3:NM_002530:exon5:c.G362T:p.R121I | 21.50% | SNP |
|  | 1810749 | 115256530 | 115256530 | G | T | NRAS | Missense_Mutation | NRAS:NM_002524:exon3:c.C181A:p.Q61K | 26.40% | SNP |
|  | 1810751 | 212488714 | 212488717 | ATAC | - | ERBB4 | Frame_Shift_Del | ERBB4:NM_001042599:exon18:c.2132_2135del:p.R711fs,ERBB4:NM_005235:exon18:c.2132_2135del:p.R711fs | 2.00% | DEL |
|  | 1810751 | 25378647 | 25378647 | T | A | KRAS | Missense_Mutation | KRAS:NM_004985:exon4:c.A351T:p.K117N,KRAS:NM_033360:exon4:c.A351T:p.K117N | 15.60% | SNP |
|  | 1810753 | 25398284 | 25398284 | C | T | KRAS | Missense_Mutation | KRAS:NM_004985:exon2:c.G35A:p.G12D,KRAS:NM_033360:exon2:c.G35A:p.G12D | 26.60% | SNP |
|  | 1810753 | 43601830 | 43601830 | G | A | RET | Missense_Mutation | RET:NM_020630:exon5:c.G874A:p.V292M,RET:NM_020975:exon5:c.G874A:p.V292M | 29.30% | SNP |
|  | 1810754 | 25398284 | 25398284 | C | T | KRAS | Missense_Mutation | KRAS:NM_004985:exon2:c.G35A:p.G12D,KRAS:NM_033360:exon2:c.G35A:p.G12D | 22.80% | SNP |
|  | 1810755 | 25398285 | 25398285 | C | T | KRAS | Missense_Mutation | KRAS:NM_004985:exon2:c.G34A:p.G12S,KRAS:NM_033360:exon2:c.G34A:p.G12S | 43.10% | SNP |
|  | 1810758 | 37881000 | 37881000 | G | C | ERBB2 | Missense_Mutation | ERBB2:NM_001289937:exon20:c.G2329C:p.V777L,ERBB2:NM_004448:exon20:c.G2329C:p.V777L,ERBB2:NM_001005862:exon23:c.G2239C:p.V747L,ERBB2:NM_001289936:exon24:c.G2284C:p.V762L | 85.80% | SNP |
|  | 1810760 | 39213219 | 39213219 | - | CA | SOS1 | Frame_Shift_Ins | SOS1:NM_005633:exon23:c.3747_3748insTG:p.P1250fs | 5.70% | INS |
|  | 1810760 | 39213220 | 39213220 | G | C | SOS1 | Missense_Mutation | SOS1:NM_005633:exon23:c.C3747G:p.F1249L | 5.60% | SNP |
|  | 1810762 | 30142872 | 30142872 | C | A | ALK | Missense_Mutation | ALK:NM_004304:exon1:c.G654T:p.Q218H | 28.30% | SNP |
|  | 1810762 | 8073583 | 8073583 | - | TCC | ERRFI1 | In_Frame_Ins | ERRFI1:NM_018948:exon4:c.1075_1076insGGA:p.V359delinsGI | 5.30% | INS |
|  | 1810763 | 66727453 | 66727453 | A | G | MAP2K1 | Missense_Mutation | MAP2K1:NM_002755:exon2:c.A169G:p.K57E | 4.40% | SNP |
|  | 1810764 | 55593648 | 55593648 | G | T | KIT | Missense_Mutation | KIT:NM_000222:exon11:c.G1714T:p.D572Y,KIT:NM_001093772:exon11:c.G1702T:p.D568Y | 2.00% | SNP |
|  | 1810766 | 25398284 | 25398284 | C | T | KRAS | Missense_Mutation | KRAS:NM_004985:exon2:c.G35A:p.G12D,KRAS:NM_033360:exon2:c.G35A:p.G12D | 56.20% | SNP |
|  | 1810766 | 43597868 | 43597868 | G | C | RET | Missense_Mutation | RET:NM_020630:exon3:c.G416C:p.W139S,RET:NM_020975:exon3:c.G416C:p.W139S | 19.00% | SNP |
|  | 1810769 | 25398281 | 25398281 | C | T | KRAS | Missense_Mutation | KRAS:NM_004985:exon2:c.G38A:p.G13D,KRAS:NM_033360:exon2:c.G38A:p.G13D | 2.80% | SNP |
|  | 1810769 | 55598105 | 55598105 | G | A | KIT | Missense_Mutation | KIT:NM_000222:exon16:c.G2302A:p.D768N,KIT:NM_001093772:exon16:c.G2290A:p.D764N | 29.10% | SNP |
|  | 1810770 | 37866641 | 37866641 | G | T | ERBB2 | Missense_Mutation | ERBB2:NM_001289937:exon7:c.G808T:p.A270S,ERBB2:NM_004448:exon7:c.G808T:p.A270S,ERBB2:NM_001005862:exon10:c.G718T:p.A240S,ERBB2:NM_001289938:exon10:c.G718T:p.A240S,ERBB2:NM_001289936:exon11:c.G763T:p.A255S | 41.60% | SNP |
|  | 1810770 | 55240807 | 55240807 | A | G | EGFR | Missense_Mutation | EGFR:NM_001346941:exon11:c.A1250G:p.Q417R,EGFR:NM_001346897:exon16:c.A1916G:p.Q639R,EGFR:NM_001346899:exon16:c.A1916G:p.Q639R,EGFR:NM_001346898:exon17:c.A2051G:p.Q684R,EGFR:NM_001346900:exon17:c.A1892G:p.Q631R,EGFR:NM_005228:exon17:c.A2051G:p.Q684R | 42.50% | SNP |
|  | 1810770 | 227660544 | 227660544 | C | T | IRS1 | Missense_Mutation | IRS1:NM_005544:exon1:c.G2911A:p.G971R | 47.20% | SNP |
|  | 1810770 | 29483047 | 29483047 | C | G | NF1 | Missense_Mutation | NF1:NM_000267:exon2:c.C107G:p.T36S,NF1:NM_001042492:exon2:c.C107G:p.T36S,NF1:NM_001128147:exon2:c.C107G:p.T36S | 51.90% | SNP |
|  | 1810773 | 29940450 | 29940450 | G | A | ALK | Nonsense_Mutation | ALK:NM_004304:exon2:c.C781T:p.R261X | 8.90% | SNP |
|  | 1810773 | 25398284 | 25398284 | C | T | KRAS | Missense_Mutation | KRAS:NM_004985:exon2:c.G35A:p.G12D,KRAS:NM_033360:exon2:c.G35A:p.G12D | 11.10% | SNP |
|  | 1810774 | 25398284 | 25398284 | C | T | KRAS | Missense_Mutation | KRAS:NM_004985:exon2:c.G35A:p.G12D,KRAS:NM_033360:exon2:c.G35A:p.G12D | 15.80% | SNP |
|  | 1810776 | 25398284 | 25398284 | C | T | KRAS | Missense_Mutation | KRAS:NM_004985:exon2:c.G35A:p.G12D,KRAS:NM_033360:exon2:c.G35A:p.G12D | 9.30% | SNP |
|  | 1810777 | 25398284 | 25398284 | C | A | KRAS | Missense_Mutation | KRAS:NM_004985:exon2:c.G35T:p.G12V,KRAS:NM_033360:exon2:c.G35T:p.G12V | 33.30% | SNP |
|  | 1810777 | 110436297 | 110436305 | CGGCGGCGG | - | IRS2 | In_Frame_Del | IRS2:NM_003749:exon1:c.2096_2104del:p.699_702del | 43.00% | DEL |
|  | 1810778 | 88472628 | 88472628 | G | T | NTRK3 | Missense_Mutation | NTRK3:NM_001320135:exon15:c.C1633A:p.Q545K,NTRK3:NM_001243101:exon16:c.C1903A:p.Q635K,NTRK3:NM_001012338:exon17:c.C1927A:p.Q643K,NTRK3:NM_002530:exon17:c.C1927A:p.Q643K | 12.00% | SNP |
|  | 1810778 | 25398284 | 25398284 | C | T | KRAS | Missense_Mutation | KRAS:NM_004985:exon2:c.G35A:p.G12D,KRAS:NM_033360:exon2:c.G35A:p.G12D | 28.40% | SNP |
|  | 1810779 | 37879881 | 37879881 | C | A | ERBB2 | Missense_Mutation | ERBB2:NM_001289937:exon18:c.C2176A:p.L726I,ERBB2:NM_004448:exon18:c.C2176A:p.L726I,ERBB2:NM_001005862:exon21:c.C2086A:p.L696I,ERBB2:NM_001289936:exon22:c.C2131A:p.L711I | 92.50% | SNP |
|  | 1810780 | 25398281 | 25398281 | C | T | KRAS | Missense_Mutation | KRAS:NM_004985:exon2:c.G38A:p.G13D,KRAS:NM_033360:exon2:c.G38A:p.G13D | 10.00% | SNP |
|  | 1810784 | 25398284 | 25398284 | C | T | KRAS | Missense_Mutation | KRAS:NM_004985:exon2:c.G35A:p.G12D,KRAS:NM_033360:exon2:c.G35A:p.G12D | 4.50% | SNP |
|  | 1810785 | 55127311 | 55127311 | - | CTCA | PDGFRA | Frame_Shift_Ins | PDGFRA:NM_001347827:exon3:c.99_100insCTCA:p.N33fs,PDGFRA:NM_001347829:exon3:c.99_100insCTCA:p.N33fs,PDGFRA:NM_001347830:exon3:c.138_139insCTCA:p.N46fs,PDGFRA:NM_006206:exon3:c.99_100insCTCA:p.N33fs,PDGFRA:NM_001347828:exon4:c.174_175insCTCA:p.N58fs | 5.70% | INS |
|  | 1810785 | 55127314 | 55127317 | AAAT | - | PDGFRA | Frame_Shift_Del | PDGFRA:NM_001347827:exon3:c.102_105del:p.E34fs,PDGFRA:NM_001347829:exon3:c.102_105del:p.E34fs,PDGFRA:NM_001347830:exon3:c.141_144del:p.E47fs,PDGFRA:NM_006206:exon3:c.102_105del:p.E34fs,PDGFRA:NM_001347828:exon4:c.177_180del:p.E59fs | 5.90% | DEL |
|  | 1810785 | 55127325 | 55127325 | T | A | PDGFRA | Missense_Mutation | PDGFRA:NM_001347827:exon3:c.T113A:p.V38D,PDGFRA:NM_001347829:exon3:c.T113A:p.V38D,PDGFRA:NM_001347830:exon3:c.T152A:p.V51D,PDGFRA:NM_006206:exon3:c.T113A:p.V38D,PDGFRA:NM_001347828:exon4:c.T188A:p.V63D | 7.60% | SNP |
|  | 1810785 | 55127349 | 55127349 | - | ATTT | PDGFRA | Frame_Shift_Ins | PDGFRA:NM_001347827:exon3:c.137_138insATTT:p.S46fs,PDGFRA:NM_001347829:exon3:c.137_138insATTT:p.S46fs,PDGFRA:NM_001347830:exon3:c.176_177insATTT:p.S59fs,PDGFRA:NM_006206:exon3:c.137_138insATTT:p.S46fs,PDGFRA:NM_001347828:exon4:c.212_213insATTT:p.S71fs | 8.90% | INS |
|  | 1810785 | 55127352 | 55127353 | TG | - | PDGFRA | Frame_Shift_Del | PDGFRA:NM_001347827:exon3:c.140_141del:p.L47fs,PDGFRA:NM_001347829:exon3:c.140_141del:p.L47fs,PDGFRA:NM_001347830:exon3:c.179_180del:p.L60fs,PDGFRA:NM_006206:exon3:c.140_141del:p.L47fs,PDGFRA:NM_001347828:exon4:c.215_216del:p.L72fs | 9.10% | DEL |
|  | 1810785 | 55127355 | 55127359 | GATGC | - | PDGFRA | Frame_Shift_Del | PDGFRA:NM_001347827:exon3:c.143_147del:p.R48fs,PDGFRA:NM_001347829:exon3:c.143_147del:p.R48fs,PDGFRA:NM_001347830:exon3:c.182_186del:p.R61fs,PDGFRA:NM_006206:exon3:c.143_147del:p.R48fs,PDGFRA:NM_001347828:exon4:c.218_222del:p.R73fs | 9.10% | DEL |
|  | 1810785 | 55127364 | 55127364 | - | AA | PDGFRA | Frame_Shift_Ins | PDGFRA:NM_001347827:exon3:c.152_153insAA:p.G51fs,PDGFRA:NM_001347829:exon3:c.152_153insAA:p.G51fs,PDGFRA:NM_001347830:exon3:c.191_192insAA:p.G64fs,PDGFRA:NM_006206:exon3:c.152_153insAA:p.G51fs,PDGFRA:NM_001347828:exon4:c.227_228insAA:p.G76fs | 8.50% | INS |
|  | 1810785 | 55127366 | 55127366 | - | AT | PDGFRA | Frame_Shift_Ins | PDGFRA:NM_001347827:exon3:c.154_155insAT:p.E52fs,PDGFRA:NM_001347829:exon3:c.154_155insAT:p.E52fs,PDGFRA:NM_001347830:exon3:c.193_194insAT:p.E65fs,PDGFRA:NM_006206:exon3:c.154_155insAT:p.E52fs,PDGFRA:NM_001347828:exon4:c.229_230insAT:p.E77fs | 8.50% | INS |
|  | 1810785 | 55131155 | 55131155 | - | CAAC | PDGFRA | Frame_Shift_Ins | PDGFRA:NM_001347827:exon5:c.698_699insCAAC:p.V233fs,PDGFRA:NM_001347829:exon5:c.698_699insCAAC:p.V233fs,PDGFRA:NM_001347830:exon5:c.737_738insCAAC:p.V246fs,PDGFRA:NM_006206:exon5:c.698_699insCAAC:p.V233fs,PDGFRA:NM_001347828:exon6:c.773_774insCAAC:p.V258fs | 5.30% | INS |
|  | 1810785 | 55131161 | 55131163 | GTG | - | PDGFRA | In_Frame_Del | PDGFRA:NM_001347827:exon5:c.704_706del:p.235_236del,PDGFRA:NM_001347829:exon5:c.704_706del:p.235_236del,PDGFRA:NM_001347830:exon5:c.743_745del:p.248_249del,PDGFRA:NM_006206:exon5:c.704_706del:p.235_236del,PDGFRA:NM_001347828:exon6:c.779_781del:p.260_261del | 5.00% | DEL |
|  | 1810785 | 55131171 | 55131171 | T | A | PDGFRA | Missense_Mutation | PDGFRA:NM_001347827:exon5:c.T714A:p.F238L,PDGFRA:NM_001347829:exon5:c.T714A:p.F238L,PDGFRA:NM_001347830:exon5:c.T753A:p.F251L,PDGFRA:NM_006206:exon5:c.T714A:p.F238L,PDGFRA:NM_001347828:exon6:c.T789A:p.F263L | 5.30% | SNP |
|  | 1810785 | 55127327 | 55127327 | - | AA | PDGFRA | Frame_Shift_Ins | PDGFRA:NM_001347827:exon3:c.115_116insAA:p.V39fs,PDGFRA:NM_001347829:exon3:c.115_116insAA:p.V39fs,PDGFRA:NM_001347830:exon3:c.154_155insAA:p.V52fs,PDGFRA:NM_006206:exon3:c.115_116insAA:p.V39fs,PDGFRA:NM_001347828:exon4:c.190_191insAA:p.V64fs | 9.10% | INS |
|  | 1810785 | 55127336 | 55127336 | A | C | PDGFRA | Missense_Mutation | PDGFRA:NM_001347827:exon3:c.A124C:p.N42H,PDGFRA:NM_001347829:exon3:c.A124C:p.N42H,PDGFRA:NM_001347830:exon3:c.A163C:p.N55H,PDGFRA:NM_006206:exon3:c.A124C:p.N42H,PDGFRA:NM_001347828:exon4:c.A199C:p.N67H | 8.50% | SNP |
|  | 1810785 | 55127338 | 55127339 | TT | - | PDGFRA | Frame_Shift_Del | PDGFRA:NM_001347827:exon3:c.126_127del:p.N42fs,PDGFRA:NM_001347829:exon3:c.126_127del:p.N42fs,PDGFRA:NM_001347830:exon3:c.165_166del:p.N55fs,PDGFRA:NM_006206:exon3:c.126_127del:p.N42fs,PDGFRA:NM_001347828:exon4:c.201_202del:p.N67fs | 8.50% | DEL |
|  | 1810785 | 55127342 | 55127342 | T | A | PDGFRA | Missense_Mutation | PDGFRA:NM_001347827:exon3:c.T130A:p.S44T,PDGFRA:NM_001347829:exon3:c.T130A:p.S44T,PDGFRA:NM_001347830:exon3:c.T169A:p.S57T,PDGFRA:NM_006206:exon3:c.T130A:p.S44T,PDGFRA:NM_001347828:exon4:c.T205A:p.S69T | 8.60% | SNP |
|  | 1810785 | 123285687 | 123285687 | - | AATGT | FGFR2 | Frame_Shift_Ins | FGFR2:NM_001320654:exon5:c.45_46insACATT:p.L16fs | 5.80% | INS |
|  | 1810785 | 123285690 | 123285690 | - | TTCC | FGFR2 | Frame_Shift_Ins | FGFR2:NM_001320654:exon5:c.42_43insGGAA:p.R15fs | 5.40% | INS |
|  | 1810785 | 123285695 | 123285695 | G | T | FGFR2 | Missense_Mutation | FGFR2:NM_001320654:exon5:c.C38A:p.A13D | 6.20% | SNP |
|  | 1810785 | 123285698 | 123285698 | A | G | FGFR2 | Missense_Mutation | FGFR2:NM_001320654:exon5:c.T35C:p.V12A | 5.00% | SNP |
|  | 1810785 | 140481417 | 140481417 | C | G | BRAF | Missense_Mutation | BRAF:NM_004333:exon11:c.G1391C:p.G464A | 2.50% | SNP |
|  | 1810785 | 140481419 | 140481419 | - | G | BRAF | Frame_Shift_Ins | BRAF:NM_004333:exon11:c.1388_1389insC:p.I463fs | 2.50% | INS |
|  | 1810785 | 140500223 | 140500223 | - | CATCT | BRAF | Frame_Shift_Ins | BRAF:NM_004333:exon7:c.918_919insAGATG:p.L307fs | 5.20% | INS |
|  | 1810785 | 140500226 | 140500226 | - | TCATCCCCTTCCGCACC | BRAF | Frame_Shift_Ins | BRAF:NM_004333:exon7:c.915_916insGGTGCGGAAGGGGATGA:p.S306fs | 5.80% | INS |
|  | 1810785 | 140500233 | 140500239 | TTCCTGT | - | BRAF | Frame_Shift_Del | BRAF:NM_004333:exon7:c.903_909del:p.P301fs | 5.20% | DEL |
|  | 1810785 | 140500241 | 140500241 | - | ACTC | BRAF | Frame_Shift_Ins | BRAF:NM_004333:exon7:c.900_901insGAGT:p.P301fs | 5.30% | INS |
|  | 1810785 | 25398284 | 25398284 | C | T | KRAS | Missense_Mutation | KRAS:NM_004985:exon2:c.G35A:p.G12D,KRAS:NM_033360:exon2:c.G35A:p.G12D | 13.90% | SNP |
|  | 1810785 | 12641248 | 12641248 | C | - | RAF1 | Frame_Shift_Del | RAF1:NM_002880:exon10:c.1050delG:p.M350fs | 6.30% | DEL |
|  | 1810785 | 12641249 | 12641249 | - | GAGAT | RAF1 | Frame_Shift_Ins | RAF1:NM_002880:exon10:c.1048_1049insATCTC:p.M350fs | 6.10% | INS |
|  | 1810785 | 12641253 | 12641256 | CTTC | - | RAF1 | Frame_Shift_Del | RAF1:NM_002880:exon10:c.1042_1045del:p.E348fs | 5.90% | DEL |
|  | 1810785 | 12641258 | 12641259 | CT | - | RAF1 | Frame_Shift_Del | RAF1:NM_002880:exon10:c.1039_1040del:p.S347fs | 6.50% | DEL |
|  | 1810785 | 12641261 | 12641264 | GCTT | - | RAF1 | Frame_Shift_Del | RAF1:NM_002880:exon10:c.1034_1037del:p.E345fs | 6.80% | DEL |
|  | 1810785 | 12641270 | 12641274 | TCCCA | - | RAF1 | Frame_Shift_Del | RAF1:NM_002880:exon10:c.1024_1028del:p.W342fs | 6.60% | DEL |
|  | 1810785 | 12641276 | 12641276 | - | TGGGA | RAF1 | Frame_Shift_Ins | RAF1:NM_002880:exon10:c.1021_1022insTCCCA:p.Y341fs | 7.00% | INS |
|  | 1810785 | 12641279 | 12641279 | - | AGA | RAF1 | In_Frame_Ins | RAF1:NM_002880:exon10:c.1018_1019insTCT:p.Y340delinsFY | 7.10% | INS |
|  | 1810785 | 12641282 | 12641282 | - | CAG | RAF1 | In_Frame_Ins | RAF1:NM_002880:exon10:c.1015_1016insCTG:p.S339delinsTG | 7.20% | INS |
|  | 1810785 | 12641283 | 12641283 | - | GAAG | RAF1 | Frame_Shift_Ins | RAF1:NM_002880:exon10:c.1014_1015insCTTC:p.S339fs | 7.10% | INS |
|  | 1810785 | 39239385 | 39239385 | G | T | SOS1 | Missense_Mutation | SOS1:NM_005633:exon14:c.C2272A:p.P758T | 5.10% | SNP |
|  | 1810785 | 39239392 | 39239392 | - | CCACAGT | SOS1 | Frame_Shift_Ins | SOS1:NM_005633:exon14:c.2264_2265insACTGTGG:p.Q755fs | 6.40% | INS |
|  | 1810785 | 39239396 | 39239398 | AAT | - | SOS1 | In_Frame_Del | SOS1:NM_005633:exon14:c.2259_2261del:p.753_754del | 5.80% | DEL |
|  | 1810785 | 39239400 | 39239400 | - | GG | SOS1 | Frame_Shift_Ins | SOS1:NM_005633:exon14:c.2256_2257insCC:p.T753fs | 5.80% | INS |
|  | 1810785 | 39239401 | 39239401 | A | C | SOS1 | Missense_Mutation | SOS1:NM_005633:exon14:c.T2256G:p.I752M | 6.20% | SNP |
|  | 1810785 | 39239406 | 39239406 | T | A | SOS1 | Missense_Mutation | SOS1:NM_005633:exon14:c.A2251T:p.N751Y | 5.30% | SNP |
|  | 1810785 | 39239407 | 39239407 | - | GC | SOS1 | Frame_Shift_Ins | SOS1:NM_005633:exon14:c.2249_2250insGC:p.H750fs | 5.40% | INS |
|  | 1810785 | 39239408 | 39239408 | T | A | SOS1 | Missense_Mutation | SOS1:NM_005633:exon14:c.A2249T:p.H750L | 5.50% | SNP |
|  | 1810785 | 55593610 | 55593610 | - | G | KIT | Frame_Shift_Ins | KIT:NM_000222:exon11:c.1676_1677insG:p.V559fs,KIT:NM_001093772:exon11:c.1664_1665insG:p.V555fs | 2.50% | INS |
|  | 1810785 | 55593616 | 55593616 | A | - | KIT | Frame_Shift_Del | KIT:NM_000222:exon11:c.1682delA:p.E561fs,KIT:NM_001093772:exon11:c.1670delA:p.E557fs | 2.50% | DEL |
|  | 1810785 | 55593648 | 55593648 | G | T | KIT | Missense_Mutation | KIT:NM_000222:exon11:c.G1714T:p.D572Y,KIT:NM_001093772:exon11:c.G1702T:p.D568Y | 2.70% | SNP |
|  | 1810785 | 8074355 | 8074358 | TTTC | - | ERRFI1 | Frame_Shift_Del | ERRFI1:NM_018948:exon4:c.301_304del:p.E101fs | 5.30% | DEL |
|  | 1810785 | 55151563 | 55151571 | AGATGATAA | - | PDGFRA | In_Frame_Del | PDGFRA:NM_001347829:exon17:c.2349_2357del:p.783_786del,PDGFRA:NM_001347830:exon17:c.2388_2396del:p.796_799del,PDGFRA:NM_006206:exon17:c.2349_2357del:p.783_786del,PDGFRA:NM_001347828:exon18:c.2424_2432del:p.808_811del | 5.10% | DEL |
|  | 1810785 | 55151575 | 55151575 | A | - | PDGFRA | Frame_Shift_Del | PDGFRA:NM_001347829:exon17:c.2361delA:p.S787fs,PDGFRA:NM_001347830:exon17:c.2400delA:p.S800fs,PDGFRA:NM_006206:exon17:c.2361delA:p.S787fs,PDGFRA:NM_001347828:exon18:c.2436delA:p.S812fs | 5.10% | DEL |
|  | 1810785 | 55151577 | 55151578 | AA | - | PDGFRA | Frame_Shift_Del | PDGFRA:NM_001347829:exon17:c.2363_2364del:p.E788fs,PDGFRA:NM_001347830:exon17:c.2402_2403del:p.E801fs,PDGFRA:NM_006206:exon17:c.2363_2364del:p.E788fs,PDGFRA:NM_001347828:exon18:c.2438_2439del:p.E813fs | 5.00% | DEL |
|  | 1810785 | 55151592 | 55151592 | T | A | PDGFRA | Nonsense_Mutation | PDGFRA:NM_001347829:exon17:c.T2378A:p.L793X,PDGFRA:NM_001347830:exon17:c.T2417A:p.L806X,PDGFRA:NM_006206:exon17:c.T2378A:p.L793X,PDGFRA:NM_001347828:exon18:c.T2453A:p.L818X | 5.30% | SNP |
|  | 1810785 | 55151602 | 55151602 | - | A | PDGFRA | Frame_Shift_Ins | PDGFRA:NM_001347829:exon17:c.2389dupA:p.L796fs,PDGFRA:NM_001347830:exon17:c.2428dupA:p.L809fs,PDGFRA:NM_006206:exon17:c.2389dupA:p.L796fs,PDGFRA:NM_001347828:exon18:c.2464dupA:p.L821fs | 5.30% | INS |
|  | 1810785 | 55151606 | 55151606 | T | - | PDGFRA | Frame_Shift_Del | PDGFRA:NM_001347829:exon17:c.2392delT:p.F798fs,PDGFRA:NM_001347830:exon17:c.2431delT:p.F811fs,PDGFRA:NM_006206:exon17:c.2392delT:p.F798fs,PDGFRA:NM_001347828:exon18:c.2467delT:p.F823fs | 5.30% | DEL |
|  | 1810785 | 55151619 | 55151619 | - | AA | PDGFRA | Frame_Shift_Ins | PDGFRA:NM_001347829:exon17:c.2405_2406insAA:p.V802fs,PDGFRA:NM_001347830:exon17:c.2444_2445insAA:p.V815fs,PDGFRA:NM_006206:exon17:c.2405_2406insAA:p.V802fs,PDGFRA:NM_001347828:exon18:c.2480_2481insAA:p.V827fs | 5.00% | INS |
|  | 1810786 | 25398254 | 25398254 | - | G | KRAS | Frame_Shift_Ins | KRAS:NM_004985:exon2:c.64dupC:p.Q22fs,KRAS:NM_033360:exon2:c.64dupC:p.Q22fs | 3.00% | INS |
|  | 1810786 | 25398262 | 25398263 | CA | - | KRAS | Frame_Shift_Del | KRAS:NM_004985:exon2:c.56_57del:p.L19fs,KRAS:NM_033360:exon2:c.56_57del:p.L19fs | 2.20% | DEL |
|  | 1810786 | 25398281 | 25398281 | C | A | KRAS | Missense_Mutation | KRAS:NM_004985:exon2:c.G38T:p.G13V,KRAS:NM_033360:exon2:c.G38T:p.G13V | 2.00% | SNP |
|  | 1810786 | 25398282 | 25398282 | C | T | KRAS | Missense_Mutation | KRAS:NM_004985:exon2:c.G37A:p.G13S,KRAS:NM_033360:exon2:c.G37A:p.G13S | 2.00% | SNP |
|  | 1810786 | 12641270 | 12641274 | TCCCA | - | RAF1 | Frame_Shift_Del | RAF1:NM_002880:exon10:c.1024_1028del:p.W342fs | 5.10% | DEL |
|  | 1810786 | 12641276 | 12641276 | - | TGGGA | RAF1 | Frame_Shift_Ins | RAF1:NM_002880:exon10:c.1021_1022insTCCCA:p.Y341fs | 6.10% | INS |
|  | 1810786 | 12641279 | 12641279 | - | AGA | RAF1 | In_Frame_Ins | RAF1:NM_002880:exon10:c.1018_1019insTCT:p.Y340delinsFY | 5.80% | INS |
|  | 1810786 | 12641282 | 12641282 | - | CAG | RAF1 | In_Frame_Ins | RAF1:NM_002880:exon10:c.1015_1016insCTG:p.S339delinsTG | 5.70% | INS |
|  | 1810786 | 12641283 | 12641283 | - | GAAG | RAF1 | Frame_Shift_Ins | RAF1:NM_002880:exon10:c.1014_1015insCTTC:p.S339fs | 5.80% | INS |
|  | 1810786 | 39239387 | 39239387 | G | C | SOS1 | Nonsense_Mutation | SOS1:NM_005633:exon14:c.C2270G:p.S757X | 5.00% | SNP |
|  | 1810786 | 39239389 | 39239389 | A | C | SOS1 | Missense_Mutation | SOS1:NM_005633:exon14:c.T2268G:p.S756R | 5.10% | SNP |
|  | 1810786 | 39239392 | 39239392 | - | CCACAGT | SOS1 | Frame_Shift_Ins | SOS1:NM_005633:exon14:c.2264_2265insACTGTGG:p.Q755fs | 6.50% | INS |
|  | 1810786 | 39239396 | 39239398 | AAT | - | SOS1 | In_Frame_Del | SOS1:NM_005633:exon14:c.2259_2261del:p.753_754del | 5.10% | DEL |
|  | 1810786 | 39239400 | 39239400 | - | GG | SOS1 | Frame_Shift_Ins | SOS1:NM_005633:exon14:c.2256_2257insCC:p.T753fs | 5.30% | INS |
|  | 1810786 | 39239401 | 39239401 | A | C | SOS1 | Missense_Mutation | SOS1:NM_005633:exon14:c.T2256G:p.I752M | 5.30% | SNP |
|  | 1810786 | 116397507 | 116397507 | G | A | MET | Missense_Mutation | MET:NM_001324402:exon6:c.G589A:p.G197S,MET:NM_000245:exon7:c.G1879A:p.G627S,MET:NM_001127500:exon7:c.G1879A:p.G627S,MET:NM_001324401:exon7:c.G1879A:p.G627S | 5.20% | SNP |
|  | 1810786 | 116397508 | 116397508 | G | T | MET | Missense_Mutation | MET:NM_001324402:exon6:c.G590T:p.G197V,MET:NM_000245:exon7:c.G1880T:p.G627V,MET:NM_001127500:exon7:c.G1880T:p.G627V,MET:NM_001324401:exon7:c.G1880T:p.G627V | 5.10% | SNP |
|  | 1810786 | 116397511 | 116397511 | C | A | MET | Missense_Mutation | MET:NM_001324402:exon6:c.C593A:p.P198H,MET:NM_000245:exon7:c.C1883A:p.P628H,MET:NM_001127500:exon7:c.C1883A:p.P628H,MET:NM_001324401:exon7:c.C1883A:p.P628H | 5.40% | SNP |
|  | 1810786 | 116397514 | 116397514 | C | G | MET | Missense_Mutation | MET:NM_001324402:exon6:c.C596G:p.A199G,MET:NM_000245:exon7:c.C1886G:p.A629G,MET:NM_001127500:exon7:c.C1886G:p.A629G,MET:NM_001324401:exon7:c.C1886G:p.A629G | 6.20% | SNP |
|  | 1810786 | 116397517 | 116397517 | T | G | MET | Missense_Mutation | MET:NM_001324402:exon6:c.T599G:p.M200R,MET:NM_000245:exon7:c.T1889G:p.M630R,MET:NM_001127500:exon7:c.T1889G:p.M630R,MET:NM_001324401:exon7:c.T1889G:p.M630R | 5.50% | SNP |
|  | 1810786 | 116397520 | 116397520 | A | C | MET | Missense_Mutation | MET:NM_001324402:exon6:c.A602C:p.N201T,MET:NM_000245:exon7:c.A1892C:p.N631T,MET:NM_001127500:exon7:c.A1892C:p.N631T,MET:NM_001324401:exon7:c.A1892C:p.N631T | 5.20% | SNP |
|  | 1810786 | 116397523 | 116397523 | - | CTGT | MET | Frame_Shift_Ins | MET:NM_001324402:exon6:c.605_606insCTGT:p.K202fs,MET:NM_000245:exon7:c.1895_1896insCTGT:p.K632fs,MET:NM_001127500:exon7:c.1895_1896insCTGT:p.K632fs,MET:NM_001324401:exon7:c.1895_1896insCTGT:p.K632fs | 6.00% | INS |
|  | 1810786 | 29559123 | 29559123 | - | GAAACTACTGC | NF1 | Frame_Shift_Ins | NF1:NM_000267:exon25:c.3230_3231insGAAACTACTGC:p.V1077fs,NF1:NM_001042492:exon25:c.3230_3231insGAAACTACTGC:p.V1077fs | 5.10% | INS |
|  | 1810786 | 12641258 | 12641259 | CT | - | RAF1 | Frame_Shift_Del | RAF1:NM_002880:exon10:c.1039_1040del:p.S347fs | 5.30% | DEL |
|  | 1810786 | 12641248 | 12641248 | C | - | RAF1 | Frame_Shift_Del | RAF1:NM_002880:exon10:c.1050delG:p.M350fs | 5.50% | DEL |
|  | 1810786 | 12641249 | 12641249 | - | GAGAT | RAF1 | Frame_Shift_Ins | RAF1:NM_002880:exon10:c.1048_1049insATCTC:p.M350fs | 5.50% | INS |
|  | 1810786 | 12641253 | 12641256 | CTTC | - | RAF1 | Frame_Shift_Del | RAF1:NM_002880:exon10:c.1042_1045del:p.E348fs | 5.00% | DEL |
|  | 1810786 | 12641261 | 12641264 | GCTT | - | RAF1 | Frame_Shift_Del | RAF1:NM_002880:exon10:c.1034_1037del:p.E345fs | 5.40% | DEL |
|  | 1810789 | 25398284 | 25398284 | C | G | KRAS | Missense_Mutation | KRAS:NM_004985:exon2:c.G35C:p.G12A,KRAS:NM_033360:exon2:c.G35C:p.G12A | 2.00% | SNP |
|  | 1810794 | 25378647 | 25378647 | T | G | KRAS | Missense_Mutation | KRAS:NM_004985:exon4:c.A351C:p.K117N,KRAS:NM_033360:exon4:c.A351C:p.K117N | 64.20% | SNP |
|  | 1810797 | 25398284 | 25398284 | C | T | KRAS | Missense_Mutation | KRAS:NM_004985:exon2:c.G35A:p.G12D,KRAS:NM_033360:exon2:c.G35A:p.G12D | 50.70% | SNP |
|  | 1810797 | 12641276 | 12641276 | - | TGGGA | RAF1 | Frame_Shift_Ins | RAF1:NM_002880:exon10:c.1021_1022insTCCCA:p.Y341fs | 5.10% | INS |
|  | 1810797 | 12641279 | 12641279 | - | AGA | RAF1 | In_Frame_Ins | RAF1:NM_002880:exon10:c.1018_1019insTCT:p.Y340delinsFY | 5.20% | INS |
|  | 1810797 | 12641283 | 12641283 | - | GAAG | RAF1 | Frame_Shift_Ins | RAF1:NM_002880:exon10:c.1014_1015insCTTC:p.S339fs | 5.00% | INS |
|  | 1810798 | 227661756 | 227661756 | G | A | IRS1 | Nonsense_Mutation | IRS1:NM_005544:exon1:c.C1699T:p.R567X | 12.70% | SNP |
|  | 1810799 | 7170626 | 7170626 | - | ATAACCCCAAACTCTG | INSR | Frame_Shift_Ins | INSR:NM_000208:exon6:c.1404_1405insCAGAGTTTGGGGTTAT:p.M469fs,INSR:NM_001079817:exon6:c.1404_1405insCAGAGTTTGGGGTTAT:p.M469fs | 5.00% | INS |
|  | 1810799 | 25380275 | 25380275 | T | G | KRAS | Missense_Mutation | KRAS:NM_004985:exon3:c.A183C:p.Q61H,KRAS:NM_033360:exon3:c.A183C:p.Q61H | 3.60% | SNP |
|  | 1870186 | 55152038 | 55152038 | G | A | PDGFRA | Missense_Mutation | PDGFRA:NM_001347829:exon18:c.G2470A:p.V824I,PDGFRA:NM_001347830:exon18:c.G2509A:p.V837I,PDGFRA:NM_006206:exon18:c.G2470A:p.V824I,PDGFRA:NM_001347828:exon19:c.G2545A:p.V849I | 42.90% | SNP |
|  | 1870186 | 110434595 | 110434595 | T | G | IRS2 | Missense_Mutation | IRS2:NM_003749:exon1:c.A3806C:p.Q1269P | 36.60% | SNP |
|  | 1870186 | 29432697 | 29432697 | C | A | ALK | Missense_Mutation | ALK:NM_004304:exon25:c.G3791T:p.R1264I | 52.00% | SNP |
|  | 1870186 | 29443670 | 29443670 | T | G | ALK | Missense_Mutation | ALK:NM_004304:exon23:c.A3547C:p.I1183L | 49.90% | SNP |
|  | 1870186 | 156823762 | 156823762 | C | T | INSRR | Missense_Mutation | INSRR:NM_014215:exon2:c.G419A:p.R140H | 47.20% | SNP |
|  | 1870188 | 56478854 | 56478854 | G | A | ERBB3 | Missense_Mutation | ERBB3:NM_001005915:exon3:c.G310A:p.V104M,ERBB3:NM_001982:exon3:c.G310A:p.V104M | 23.10% | SNP |
|  | 1870188 | 533329 | 533329 | G | A | HRAS | Missense_Mutation | HRAS:NM_001318054:exon5:c.C161T:p.P54L | 26.90% | SNP |
|  | 1870188 | 7170598 | 7170598 | C | T | INSR | Missense_Mutation | INSR:NM_000208:exon6:c.G1433A:p.R478H,INSR:NM_001079817:exon6:c.G1433A:p.R478H | 21.90% | SNP |
|  | 1870188 | 29443612 | 29443612 | C | - | ALK | Frame_Shift_Del | ALK:NM_004304:exon23:c.3605delG:p.G1202fs | 22.00% | DEL |
|  | 1870189 | 56493492 | 56493492 | G | A | ERBB3 | Missense_Mutation | ERBB3:NM_001982:exon24:c.G2900A:p.R967K | 39.20% | SNP |
|  | 1870189 | 156823805 | 156823805 | C | T | INSRR | Missense_Mutation | INSRR:NM_014215:exon2:c.G376A:p.V126M | 41.30% | SNP |
|  | 1870201 | 39239450 | 39239450 | A | C | SOS1 | Missense_Mutation | SOS1:NM_005633:exon14:c.T2207G:p.I736R | 28.50% | SNP |
|  | 1870202 | 25398284 | 25398284 | C | A | KRAS | Missense_Mutation | KRAS:NM_004985:exon2:c.G35T:p.G12V,KRAS:NM_033360:exon2:c.G35T:p.G12V | 7.90% | SNP |
|  | 1870205 | 123239453 | 123239453 | T | A | FGFR2 | Missense_Mutation | FGFR2:NM_001144914:exon15:c.A2048T:p.D683V,FGFR2:NM_001144916:exon15:c.A2039T:p.D680V,FGFR2:NM_001144917:exon16:c.A2036T:p.D679V,FGFR2:NM_001144918:exon16:c.A2033T:p.D678V,FGFR2:NM_023029:exon16:c.A2117T:p.D706V,FGFR2:NM_001320654:exon17:c.A1700T:p.D567V,FGFR2:NM_000141:exon18:c.A2384T:p.D795V,FGFR2:NM_001320658:exon18:c.A2378T:p.D793V,FGFR2:NM_022970:exon18:c.A2387T:p.D796V | 5.00% | SNP |
|  | 1870205 | 25380276 | 25380276 | T | A | KRAS | Missense_Mutation | KRAS:NM_004985:exon3:c.A182T:p.Q61L,KRAS:NM_033360:exon3:c.A182T:p.Q61L | 55.70% | SNP |
|  | 1870206 | 227663174 | 227663174 | G | A | IRS1 | Missense_Mutation | IRS1:NM_005544:exon1:c.C281T:p.A94V | 5.40% | SNP |
|  | 1870206 | 55524224 | 55524224 | C | T | KIT | Missense_Mutation | KIT:NM_000222:exon1:c.C43T:p.L15F,KIT:NM_001093772:exon1:c.C43T:p.L15F | 5.30% | SNP |
|  | 1870206 | 25378562 | 25378562 | C | T | KRAS | Missense_Mutation | KRAS:NM_004985:exon4:c.G436A:p.A146T,KRAS:NM_033360:exon4:c.G436A:p.A146T | 5.70% | SNP |
|  | 1870206 | 29560048 | 29560049 | AA | - | NF1 | Frame_Shift_Del | NF1:NM_000267:exon27:c.3525_3526del:p.T1175fs,NF1:NM_001042492:exon27:c.3525_3526del:p.T1175fs | 5.70% | DEL |
|  | 1870207 | 25398281 | 25398281 | C | T | KRAS | Missense_Mutation | KRAS:NM_004985:exon2:c.G38A:p.G13D,KRAS:NM_033360:exon2:c.G38A:p.G13D | 19.60% | SNP |
|  | 1870209 | 25398285 | 25398285 | C | T | KRAS | Missense_Mutation | KRAS:NM_004985:exon2:c.G34A:p.G12S,KRAS:NM_033360:exon2:c.G34A:p.G12S | 42.50% | SNP |
|  | 1870210 | 25398281 | 25398281 | C | T | KRAS | Missense_Mutation | KRAS:NM_004985:exon2:c.G38A:p.G13D,KRAS:NM_033360:exon2:c.G38A:p.G13D | 58.90% | SNP |
|  | 1870210 | 56487287 | 56487287 | C | T | ERBB3 | Missense_Mutation | ERBB3:NM_001982:exon12:c.C1433T:p.T478M | 11.80% | SNP |
|  | 1870210 | 110437257 | 110437257 | G | C | IRS2 | Missense_Mutation | IRS2:NM_003749:exon1:c.C1144G:p.P382A | 19.00% | SNP |
|  | 1870211 | 25398284 | 25398284 | C | T | KRAS | Missense_Mutation | KRAS:NM_004985:exon2:c.G35A:p.G12D,KRAS:NM_033360:exon2:c.G35A:p.G12D | 6.20% | SNP |
|  | 1870212 | 25398284 | 25398284 | C | T | KRAS | Missense_Mutation | KRAS:NM_004985:exon2:c.G35A:p.G12D,KRAS:NM_033360:exon2:c.G35A:p.G12D | 21.60% | SNP |
|  | 1870215 | 25398284 | 25398284 | C | A | KRAS | Missense_Mutation | KRAS:NM_004985:exon2:c.G35T:p.G12V,KRAS:NM_033360:exon2:c.G35T:p.G12V | 46.60% | SNP |
|  | 1870219 | 116371873 | 116371873 | C | T | MET | Missense_Mutation | MET:NM_001324402:exon2:c.C62T:p.T21I,MET:NM_000245:exon3:c.C1352T:p.T451I,MET:NM_001127500:exon3:c.C1352T:p.T451I,MET:NM_001324401:exon3:c.C1352T:p.T451I | 27.80% | SNP |
|  | 1870220 | 56481922 | 56481922 | G | A | ERBB3 | Missense_Mutation | ERBB3:NM_001982:exon7:c.G850A:p.G284R | 50.10% | SNP |
|  | 1870221 | 56478928 | 56478928 | C | G | ERBB3 | Missense_Mutation | ERBB3:NM_001005915:exon3:c.C384G:p.S128R,ERBB3:NM_001982:exon3:c.C384G:p.S128R | 54.00% | SNP |
|  | 1870222 | 25398284 | 25398284 | C | T | KRAS | Missense_Mutation | KRAS:NM_004985:exon2:c.G35A:p.G12D,KRAS:NM_033360:exon2:c.G35A:p.G12D | 13.00% | SNP |
|  | 1870223 | 140481402 | 140481402 | C | G | BRAF | Missense_Mutation | BRAF:NM_004333:exon11:c.G1406C:p.G469A | 26.30% | SNP |
|  | 1870225 | 212483924 | 212483924 | T | C | ERBB4 | Missense_Mutation | ERBB4:NM_001042599:exon19:c.A2279G:p.K760R,ERBB4:NM_005235:exon19:c.A2279G:p.K760R | 12.30% | SNP |
|  | 1870229 | 99452048 | 99452048 | G | A | IGF1R | Missense_Mutation | IGF1R:NM_000875:exon6:c.G1382A:p.R461H,IGF1R:NM_001291858:exon6:c.G1382A:p.R461H | 16.70% | SNP |
|  | 1870229 | 110435345 | 110435345 | G | - | IRS2 | Frame_Shift_Del | IRS2:NM_003749:exon1:c.3056delC:p.P1019fs | 10.90% | DEL |
|  | 1870229 | 7170598 | 7170598 | C | T | INSR | Missense_Mutation | INSR:NM_000208:exon6:c.G1433A:p.R478H,INSR:NM_001079817:exon6:c.G1433A:p.R478H | 5.50% | SNP |
|  | 1870229 | 1807582 | 1807582 | C | T | FGFR3 | Missense_Mutation | FGFR3:NM_022965:exon11:c.C1415T:p.P472L,FGFR3:NM_000142:exon13:c.C1751T:p.P584L,FGFR3:NM_001163213:exon13:c.C1757T:p.P586L | 15.20% | SNP |
|  | 1870229 | 140534476 | 140534476 | C | T | BRAF | Missense_Mutation | BRAF:NM_004333:exon3:c.G437A:p.R146Q | 9.60% | SNP |
|  | 1870230 | 117647420 | 117647420 | T | G | ROS1 | Missense_Mutation | ROS1:NM_002944:exon33:c.A5524C:p.S1842R | 26.20% | SNP |
|  | 1870233 | 86659245 | 86659245 | C | T | RASA1 | Nonsense_Mutation | RASA1:NM_002890:exon11:c.C1534T:p.R512X,RASA1:NM_022650:exon11:c.C1003T:p.R335X | 25.20% | SNP |
|  | 1870234 | 25398284 | 25398284 | C | T | KRAS | Missense_Mutation | KRAS:NM_004985:exon2:c.G35A:p.G12D,KRAS:NM_033360:exon2:c.G35A:p.G12D | 10.10% | SNP |
|  | 1870234 | 123353268 | 123353268 | G | A | FGFR2 | Missense_Mutation | FGFR2:NM_001144913:exon1:c.C64T:p.R22W,FGFR2:NM_001144914:exon1:c.C64T:p.R22W,FGFR2:NM_001144916:exon1:c.C64T:p.R22W,FGFR2:NM_023029:exon1:c.C64T:p.R22W,FGFR2:NM_000141:exon2:c.C64T:p.R22W,FGFR2:NM_001144915:exon2:c.C64T:p.R22W,FGFR2:NM_001144917:exon2:c.C64T:p.R22W,FGFR2:NM_001144918:exon2:c.C64T:p.R22W,FGFR2:NM_001144919:exon2:c.C64T:p.R22W,FGFR2:NM_001320658:exon2:c.C64T:p.R22W,FGFR2:NM_022970:exon2:c.C64T:p.R22W | 12.90% | SNP |
|  | 1870238 | 37868208 | 37868208 | C | T | ERBB2 | Missense_Mutation | ERBB2:NM_001289937:exon8:c.C929T:p.S310F,ERBB2:NM_004448:exon8:c.C929T:p.S310F,ERBB2:NM_001005862:exon11:c.C839T:p.S280F,ERBB2:NM_001289938:exon11:c.C839T:p.S280F,ERBB2:NM_001289936:exon12:c.C884T:p.S295F | 94.70% | SNP |
|  | 1870244 | 25398284 | 25398284 | C | T | KRAS | Missense_Mutation | KRAS:NM_004985:exon2:c.G35A:p.G12D,KRAS:NM_033360:exon2:c.G35A:p.G12D | 9.70% | SNP |
|  | 1870245 | 534285 | 534285 | C | T | HRAS | Missense_Mutation | HRAS:NM_001130442:exon2:c.G38A:p.G13D,HRAS:NM_005343:exon2:c.G38A:p.G13D,HRAS:NM_176795:exon2:c.G38A:p.G13D | 35.30% | SNP |
|  | 1870246 | 25398284 | 25398284 | C | T | KRAS | Missense_Mutation | KRAS:NM_004985:exon2:c.G35A:p.G12D,KRAS:NM_033360:exon2:c.G35A:p.G12D | 8.60% | SNP |
|  | 1870251 | 22142984 | 22142984 | C | A | MAPK1 | Missense_Mutation | MAPK1:NM_002745:exon5:c.G723T:p.L241F,MAPK1:NM_138957:exon5:c.G723T:p.L241F | 23.40% | SNP |
|  | 1870253 | 25398284 | 25398284 | C | A | KRAS | Missense_Mutation | KRAS:NM_004985:exon2:c.G35T:p.G12V,KRAS:NM_033360:exon2:c.G35T:p.G12V | 18.40% | SNP |
|  | 1870254 | 532660 | 532660 | C | T | HRAS | Translation_Start_Site | HRAS:NM_001130442:exon5:c.G546A:p.M182I,HRAS:NM_005343:exon5:c.G546A:p.M182I,HRAS:NM_001318054:exon6:c.G309A:p.M103I | 35.10% | SNP |
|  | 1870255 | 25398285 | 25398285 | C | T | KRAS | Missense_Mutation | KRAS:NM_004985:exon2:c.G34A:p.G12S,KRAS:NM_033360:exon2:c.G34A:p.G12S | 4.70% | SNP |
|  | 1870255 | 112926888 | 112926888 | G | C | PTPN11 | Missense_Mutation | PTPN11:NM_001330437:exon13:c.G1520C:p.G507A,PTPN11:NM_002834:exon13:c.G1508C:p.G503A | 3.70% | SNP |
|  | 1870258 | 25398281 | 25398281 | C | T | KRAS | Missense_Mutation | KRAS:NM_004985:exon2:c.G38A:p.G13D,KRAS:NM_033360:exon2:c.G38A:p.G13D | 38.40% | SNP |
|  | 1870262 | 156844722 | 156844722 | G | A | NTRK1 | Missense_Mutation | NTRK1:NM_001012331:exon10:c.G1258A:p.V420I,NTRK1:NM_001007792:exon11:c.G1168A:p.V390I,NTRK1:NM_002529:exon11:c.G1276A:p.V426I | 25.90% | SNP |
|  | 1870264 | 25380282 | 25380282 | G | T | KRAS | Missense_Mutation | KRAS:NM_004985:exon3:c.C176A:p.A59E,KRAS:NM_033360:exon3:c.C176A:p.A59E | 2.50% | SNP |
|  | 1870264 | 140481411 | 140481411 | C | A | BRAF | Missense_Mutation | BRAF:NM_004333:exon11:c.G1397T:p.G466V | 5.10% | SNP |
|  | 1870264 | 140481412 | 140481412 | C | A | BRAF | Nonsense_Mutation | BRAF:NM_004333:exon11:c.G1396T:p.G466X | 5.10% | SNP |
|  | 1870265 | 29559872 | 29559872 | G | A | NF1 | Missense_Mutation | NF1:NM_000267:exon26:c.G3469A:p.V1157I,NF1:NM_001042492:exon26:c.G3469A:p.V1157I | 17.40% | SNP |
|  | 1870265 | 25398284 | 25398284 | C | G | KRAS | Missense_Mutation | KRAS:NM_004985:exon2:c.G35C:p.G12A,KRAS:NM_033360:exon2:c.G35C:p.G12A | 34.00% | SNP |
|  | 1870267 | 25378562 | 25378562 | C | T | KRAS | Missense_Mutation | KRAS:NM_004985:exon4:c.G436A:p.A146T,KRAS:NM_033360:exon4:c.G436A:p.A146T | 19.90% | SNP |
|  | 1870268 | 25398284 | 25398284 | C | G | KRAS | Missense_Mutation | KRAS:NM_004985:exon2:c.G35C:p.G12A,KRAS:NM_033360:exon2:c.G35C:p.G12A | 30.20% | SNP |
|  | 1870270 | 55268077 | 55268077 | C | T | EGFR | Nonsense_Mutation | EGFR:NM_001346941:exon18:c.C2116T:p.R706X,EGFR:NM_001346897:exon23:c.C2782T:p.R928X,EGFR:NM_001346899:exon23:c.C2782T:p.R928X,EGFR:NM_001346898:exon24:c.C2917T:p.R973X,EGFR:NM_001346900:exon24:c.C2758T:p.R920X,EGFR:NM_005228:exon24:c.C2917T:p.R973X | 22.70% | SNP |
|  | 1870272 | 38273472 | 38273472 | G | T | FGFR1 | Missense_Mutation | FGFR1:NM_001174066:exon12:c.C1503A:p.N501K,FGFR1:NM_023105:exon12:c.C1503A:p.N501K,FGFR1:NM_023106:exon12:c.C1497A:p.N499K,FGFR1:NM_001174063:exon13:c.C1764A:p.N588K,FGFR1:NM_001174065:exon13:c.C1764A:p.N588K,FGFR1:NM_015850:exon13:c.C1764A:p.N588K,FGFR1:NM_023110:exon13:c.C1770A:p.N590K,FGFR1:NM_001174064:exon14:c.C1740A:p.N580K,FGFR1:NM_001174067:exon14:c.C1863A:p.N621K | 16.80% | SNP |
|  | 1870272 | 112924315 | 112924315 | C | T | PTPN11 | Missense_Mutation | PTPN11:NM_001330437:exon11:c.C1273T:p.R425W,PTPN11:NM_002834:exon11:c.C1261T:p.R421W,PTPN11:NM_080601:exon11:c.C1261T:p.R421W | 16.70% | SNP |
|  | 1870272 | 25398281 | 25398281 | C | T | KRAS | Missense_Mutation | KRAS:NM_004985:exon2:c.G38A:p.G13D,KRAS:NM_033360:exon2:c.G38A:p.G13D | 16.20% | SNP |
|  | 1870273 | 88476335 | 88476335 | G | T | NTRK3 | Missense_Mutation | NTRK3:NM_001320135:exon14:c.C1503A:p.H501Q,NTRK3:NM_001243101:exon15:c.C1773A:p.H591Q,NTRK3:NM_001012338:exon16:c.C1797A:p.H599Q,NTRK3:NM_002530:exon16:c.C1797A:p.H599Q | 14.80% | SNP |
|  | 1870273 | 39213438 | 39213438 | C | T | SOS1 | Missense_Mutation | SOS1:NM_005633:exon23:c.G3529A:p.D1177N | 12.50% | SNP |
|  | 1870276 | 117609935 | 117609935 | G | C | ROS1 | Nonsense_Mutation | ROS1:NM_002944:exon43:c.C6764G:p.S2255X | 29.10% | SNP |
|  | 1870281 | 25398284 | 25398284 | C | A | KRAS | Missense_Mutation | KRAS:NM_004985:exon2:c.G35T:p.G12V,KRAS:NM_033360:exon2:c.G35T:p.G12V | 39.00% | SNP |
|  | 1870282 | 47425446 | 47425446 | C | T | ARHGAP35 | Missense_Mutation | ARHGAP35:NM_004491:exon1:c.C3514T:p.R1172W | 5.80% | SNP |
|  | 1870283 | 115258748 | 115258748 | C | A | NRAS | Missense_Mutation | NRAS:NM_002524:exon2:c.G34T:p.G12C | 6.80% | SNP |
|  | 1870284 | 25398284 | 25398284 | C | T | KRAS | Missense_Mutation | KRAS:NM_004985:exon2:c.G35A:p.G12D,KRAS:NM_033360:exon2:c.G35A:p.G12D | 19.40% | SNP |
|  | 1870285 | 29432662 | 29432662 | C | A | ALK | Missense_Mutation | ALK:NM_004304:exon25:c.G3826T:p.D1276Y | 17.80% | SNP |
|  | 1870285 | 43596003 | 43596003 | G | A | RET | Missense_Mutation | RET:NM_020630:exon2:c.G170A:p.R57Q,RET:NM_020975:exon2:c.G170A:p.R57Q | 19.30% | SNP |
|  | 1870285 | 43601837 | 43601837 | C | T | RET | Missense_Mutation | RET:NM_020630:exon5:c.C881T:p.A294V,RET:NM_020975:exon5:c.C881T:p.A294V | 18.70% | SNP |
|  | 1870285 | 7267564 | 7267564 | C | G | INSR | Missense_Mutation | INSR:NM_000208:exon2:c.G444C:p.K148N,INSR:NM_001079817:exon2:c.G444C:p.K148N | 6.10% | SNP |
|  | 1870285 | 7267787 | 7267787 | C | T | INSR | Missense_Mutation | INSR:NM_000208:exon2:c.G221A:p.R74Q,INSR:NM_001079817:exon2:c.G221A:p.R74Q | 17.30% | SNP |
|  | 1870285 | 25378562 | 25378562 | C | T | KRAS | Missense_Mutation | KRAS:NM_004985:exon4:c.G436A:p.A146T,KRAS:NM_033360:exon4:c.G436A:p.A146T | 18.10% | SNP |
|  | 1870286 | 55214430 | 55214436 | AGCTGTA | - | EGFR | Frame_Shift_Del | EGFR:NM_001346898:exon4:c.556_559del:p.S186fs,EGFR:NM_001346900:exon4:c.397_400del:p.S133fs,EGFR:NM_005228:exon4:c.556_559del:p.S186fs,EGFR:NM_201282:exon4:c.556_559del:p.S186fs,EGFR:NM_201283:exon4:c.556_559del:p.S186fs,EGFR:NM_201284:exon4:c.556_559del:p.S186fs | 14.50% | DEL |
|  | 1870288 | 25398284 | 25398284 | C | A | KRAS | Missense_Mutation | KRAS:NM_004985:exon2:c.G35T:p.G12V,KRAS:NM_033360:exon2:c.G35T:p.G12V | 12.10% | SNP |
|  | 1870288 | 12641248 | 12641248 | C | - | RAF1 | Frame_Shift_Del | RAF1:NM_002880:exon10:c.1050delG:p.M350fs | 5.20% | DEL |
|  | 1870288 | 12641249 | 12641249 | - | GAGAT | RAF1 | Frame_Shift_Ins | RAF1:NM_002880:exon10:c.1048_1049insATCTC:p.M350fs | 5.10% | INS |
|  | 1870291 | 25398285 | 25398285 | C | T | KRAS | Missense_Mutation | KRAS:NM_004985:exon2:c.G34A:p.G12S,KRAS:NM_033360:exon2:c.G34A:p.G12S | 39.60% | SNP |
|  | 1870293 | 55593610 | 55593610 | - | G | KIT | Frame_Shift_Ins | KIT:NM_000222:exon11:c.1676_1677insG:p.V559fs,KIT:NM_001093772:exon11:c.1664_1665insG:p.V555fs | 2.00% | INS |
|  | 1870295 | 25398281 | 25398281 | C | T | KRAS | Missense_Mutation | KRAS:NM_004985:exon2:c.G38A:p.G13D,KRAS:NM_033360:exon2:c.G38A:p.G13D | 3.60% | SNP |
|  | 1870298 | 25398284 | 25398284 | C | T | KRAS | Missense_Mutation | KRAS:NM_004985:exon2:c.G35A:p.G12D,KRAS:NM_033360:exon2:c.G35A:p.G12D | 24.90% | SNP |
|  | 1870299 | 176520717 | 176520717 | T | G | FGFR4 | Missense_Mutation | FGFR4:NM_022963:exon9:c.T1340G:p.F447C,FGFR4:NM_001291980:exon11:c.T1256G:p.F419C,FGFR4:NM_002011:exon11:c.T1460G:p.F487C,FGFR4:NM_213647:exon11:c.T1460G:p.F487C | 19.10% | SNP |
|  | 1870300 | 25398284 | 25398284 | C | T | KRAS | Missense_Mutation | KRAS:NM_004985:exon2:c.G35A:p.G12D,KRAS:NM_033360:exon2:c.G35A:p.G12D | 33.30% | SNP |
|  | 1870300 | 116339258 | 116339258 | G | T | MET | Missense_Mutation | MET:NM_000245:exon2:c.G120T:p.K40N,MET:NM_001127500:exon2:c.G120T:p.K40N,MET:NM_001324401:exon2:c.G120T:p.K40N | 16.80% | SNP |
|  | 1870300 | 43622077 | 43622077 | G | A | RET | Missense_Mutation | RET:NM_020630:exon19:c.G3094A:p.G1032S,RET:NM_020975:exon19:c.G3094A:p.G1032S | 15.20% | SNP |
| Smad | 1810382 | 48573428 | 48573428 | G | A | SMAD4 | Missense_Mutation | SMAD4:NM_005359:exon2:c.G12A:p.M4I | 30.40% | SNP |
|  | 1810383 | 48591919 | 48591919 | G | A | SMAD4 | Missense_Mutation | SMAD4:NM_005359:exon9:c.G1082A:p.R361H | 41.30% | SNP |
|  | 1810391 | 45374932 | 45374932 | T | C | SMAD2 | Missense_Mutation | SMAD2:NM_001135937:exon7:c.A821G:p.D274G,SMAD2:NM_001003652:exon8:c.A911G:p.D304G,SMAD2:NM_005901:exon8:c.A911G:p.D304G | 20.80% | SNP |
|  | 1810391 | 67477202 | 67477202 | G | - | SMAD3 | Frame_Shift_Del | SMAD3:NM_001145104:exon5:c.424delG:p.G142fs,SMAD3:NM_001145102:exon7:c.694delG:p.G232fs,SMAD3:NM_001145103:exon7:c.877delG:p.G293fs,SMAD3:NM_005902:exon7:c.1009delG:p.G337fs | 18.30% | DEL |
|  | 1810727 | 48604754 | 48604754 | G | A | SMAD4 | Missense_Mutation | SMAD4:NM_005359:exon12:c.G1576A:p.E526K | 22.50% | SNP |
|  | 1810730 | 67358568 | 67358568 | C | T | SMAD3 | Nonsense_Mutation | SMAD3:NM_005902:exon1:c.C76T:p.Q26X | 11.30% | SNP |
|  | 1810737 | 48575063 | 48575063 | G | T | SMAD4 | Missense_Mutation | SMAD4:NM_005359:exon3:c.G257T:p.G86V | 18.30% | SNP |
|  | 1810744 | 48603053 | 48603053 | G | A | SMAD4 | Missense_Mutation | SMAD4:NM_005359:exon11:c.G1354A:p.A452T | 55.20% | SNP |
|  | 1810745 | 48573545 | 48573545 | G | C | SMAD4 | Missense_Mutation | SMAD4:NM_005359:exon2:c.G129C:p.L43F | 59.80% | SNP |
|  | 1810748 | 48603009 | 48603009 | T | A | SMAD4 | Missense_Mutation | SMAD4:NM_005359:exon11:c.T1310A:p.V437D | 6.00% | SNP |
|  | 1810753 | 48575132 | 48575132 | T | C | SMAD4 | Missense_Mutation | SMAD4:NM_005359:exon3:c.T326C:p.L109P | 39.00% | SNP |
|  | 1810754 | 45371713 | 45371713 | G | C | SMAD2 | Nonsense_Mutation | SMAD2:NM_001135937:exon9:c.C1188G:p.Y396X,SMAD2:NM_001003652:exon10:c.C1278G:p.Y426X,SMAD2:NM_005901:exon10:c.C1278G:p.Y426X | 39.20% | SNP |
|  | 1810760 | 45374906 | 45374906 | A | T | SMAD2 | Missense_Mutation | SMAD2:NM_001135937:exon7:c.T847A:p.L283I,SMAD2:NM_001003652:exon8:c.T937A:p.L313I,SMAD2:NM_005901:exon8:c.T937A:p.L313I | 34.10% | SNP |
|  | 1810760 | 67457403 | 67457403 | A | G | SMAD3 | Missense_Mutation | SMAD3:NM_001145102:exon2:c.A62G:p.H21R,SMAD3:NM_001145103:exon2:c.A245G:p.H82R,SMAD3:NM_005902:exon2:c.A377G:p.H126R | 39.00% | SNP |
|  | 1810790 | 67473779 | 67473779 | C | T | SMAD3 | Missense_Mutation | SMAD3:NM_001145104:exon4:c.C274T:p.R92W,SMAD3:NM_001145102:exon6:c.C544T:p.R182W,SMAD3:NM_001145103:exon6:c.C727T:p.R243W,SMAD3:NM_005902:exon6:c.C859T:p.R287W | 50.50% | SNP |
|  | 1810791 | 48603035 | 48603035 | C | T | SMAD4 | Nonsense_Mutation | SMAD4:NM_005359:exon11:c.C1336T:p.Q446X | 10.40% | SNP |
|  | 1810794 | 45394798 | 45394799 | GT | - | SMAD2 | Frame_Shift_Del | SMAD2:NM_001135937:exon4:c.460_461del:p.T154fs,SMAD2:NM_001003652:exon5:c.550_551del:p.T184fs,SMAD2:NM_005901:exon5:c.550_551del:p.T184fs | 56.80% | DEL |
|  | 1810794 | 48604750 | 48604750 | G | A | SMAD4 | Nonsense_Mutation | SMAD4:NM_005359:exon12:c.G1572A:p.W524X | 56.10% | SNP |
|  | 1810797 | 48593463 | 48593463 | - | CGCG | SMAD4 | Frame_Shift_Ins | SMAD4:NM_005359:exon10:c.1214_1215insCGCG:p.H405fs | 60.60% | INS |
|  | 1870207 | 48573522 | 48573530 | GCAAAAAGA | - | SMAD4 | In_Frame_Del | SMAD4:NM_005359:exon2:c.106_114del:p.36_38del | 10.40% | DEL |
|  | 1870212 | 45368211 | 45368211 | G | A | SMAD2 | Missense_Mutation | SMAD2:NM_001135937:exon10:c.C1301T:p.S434L,SMAD2:NM_001003652:exon11:c.C1391T:p.S464L,SMAD2:NM_005901:exon11:c.C1391T:p.S464L | 23.30% | SNP |
|  | 1870212 | 48593466 | 48593466 | C | T | SMAD4 | Missense_Mutation | SMAD4:NM_005359:exon10:c.C1217T:p.A406V | 19.80% | SNP |
|  | 1870217 | 45374938 | 45374938 | A | C | SMAD2 | Missense_Mutation | SMAD2:NM_001135937:exon7:c.T815G:p.F272C,SMAD2:NM_001003652:exon8:c.T905G:p.F302C,SMAD2:NM_005901:exon8:c.T905G:p.F302C | 34.80% | SNP |
|  | 1870221 | 48591925 | 48591925 | G | C | SMAD4 | Missense_Mutation | SMAD4:NM_005359:exon9:c.G1088C:p.C363S | 63.20% | SNP |
|  | 1870223 | 48575209 | 48575209 | C | T | SMAD4 | Nonsense_Mutation | SMAD4:NM_005359:exon3:c.C403T:p.R135X | 38.80% | SNP |
|  | 1870229 | 48591919 | 48591919 | G | A | SMAD4 | Missense_Mutation | SMAD4:NM_005359:exon9:c.G1082A:p.R361H | 10.30% | SNP |
|  | 1870236 | 48593406 | 48593406 | G | T | SMAD4 | Missense_Mutation | SMAD4:NM_005359:exon10:c.G1157T:p.G386V | 22.10% | SNP |
|  | 1870240 | 67457361 | 67457361 | C | T | SMAD3 | Missense_Mutation | SMAD3:NM_001145102:exon2:c.C20T:p.A7V,SMAD3:NM_001145103:exon2:c.C203T:p.A68V,SMAD3:NM_005902:exon2:c.C335T:p.A112V | 10.10% | SNP |
|  | 1870242 | 67482861 | 67482861 | C | - | SMAD3 | Frame_Shift_Del | SMAD3:NM_001145104:exon7:c.680delC:p.S227fs,SMAD3:NM_001145102:exon9:c.950delC:p.S317fs,SMAD3:NM_001145103:exon9:c.1133delC:p.S378fs,SMAD3:NM_005902:exon9:c.1265delC:p.S422fs | 15.40% | DEL |
|  | 1870245 | 45395639 | 45395639 | - | T | SMAD2 | Nonsense_Mutation | SMAD2:NM_001135937:exon3:c.404dupA:p.Y135_H136delinsX,SMAD2:NM_001003652:exon4:c.494dupA:p.Y165_H166delinsX,SMAD2:NM_005901:exon4:c.494dupA:p.Y165_H166delinsX | 59.50% | INS |
|  | 1870245 | 67457310 | 67457310 | C | T | SMAD3 | Missense_Mutation | SMAD3:NM_001145103:exon2:c.C152T:p.P51L,SMAD3:NM_005902:exon2:c.C284T:p.P95L | 69.60% | SNP |
|  | 1870253 | 48584594 | 48584594 | - | G | SMAD4 | Frame_Shift_Ins | SMAD4:NM_005359:exon6:c.768dupG:p.Q256fs | 21.50% | INS |
|  | 1870258 | 48591919 | 48591919 | G | A | SMAD4 | Missense_Mutation | SMAD4:NM_005359:exon9:c.G1082A:p.R361H | 39.00% | SNP |
|  | 1870260 | 48591918 | 48591918 | C | T | SMAD4 | Missense_Mutation | SMAD4:NM_005359:exon9:c.C1081T:p.R361C | 21.00% | SNP |
|  | 1870262 | 48593491 | 48593494 | AGAC | - | SMAD4 | Frame_Shift_Del | SMAD4:NM_005359:exon10:c.1242_1245del:p.L414fs | 44.60% | DEL |
|  | 1870267 | 67482873 | 67482873 | A | C | SMAD3 | Nonstop_Mutation | SMAD3:NM_001145104:exon7:c.A692C:p.X231S,SMAD3:NM_001145102:exon9:c.A962C:p.X321S,SMAD3:NM_001145103:exon9:c.A1145C:p.X382S,SMAD3:NM_005902:exon9:c.A1277C:p.X426S | 23.50% | SNP |
|  | 1870270 | 48575182 | 48575182 | G | T | SMAD4 | Missense_Mutation | SMAD4:NM_005359:exon3:c.G376T:p.V126F | 48.20% | SNP |
|  | 1870271 | 48575103 | 48575103 | G | C | SMAD4 | Missense_Mutation | SMAD4:NM_005359:exon3:c.G297C:p.W99C | 10.90% | SNP |
|  | 1870281 | 48593411 | 48593411 | C | T | SMAD4 | Nonsense_Mutation | SMAD4:NM_005359:exon10:c.C1162T:p.Q388X | 27.10% | SNP |
|  | 1870285 | 48591837 | 48591837 | C | T | SMAD4 | Nonsense_Mutation | SMAD4:NM_005359:exon9:c.C1000T:p.Q334X | 12.30% | SNP |
