# Supplementary material for: Characterization of Genomic Alterations in Colorectal Liver Metastasis and Their Prognostic Value
Source: Front Cell Dev Biol. 2022 Jul 4;9:760618. doi: 10.3389/fcell.2021.760618 (PMC9289210; doi:10.3389/fcell.2021.760618)
Supplement: Supplementary file 8 [file Table6.DOCX]

| Gene | Mutation | Left side  n (%) | Right side  n (%) | *p* value |
| --- | --- | --- | --- | --- |
| KRAS | 62 |  |  | 0.043089 |
| Mutation |  | 47 (39%) | 15 (63%) |  |
| WT |  | 73 (61%) | 9 (38%) |  |
| AMER1 | 15 |  |  | 0.020477 |
| Mutation |  | 9 (8%) | 6 (25%) |  |
| WT |  | 111 (93%) | 18 (75%) |  |
| NSD1 | 7 |  |  | 0.015041 |
| Mutation |  | 3 (3%) | 4 (17%) |  |
| WT |  | 117 (98%) | 20 (83%) |  |
| EPPK1 | 7 |  |  | 0.015041 |
| Mutation |  | 3 (3%) | 4 (17%) |  |
| WT |  | 117 (98%) | 20 (83%) |  |
| PIK3R1 | 5 |  |  | 0.002739 |
| Mutation |  | 1 (1%) | 4 (17%) |  |
| WT |  | 119 (99%) | 20 (83%) |  |
| ACVR2A | 5 |  |  | 0.032783 |
| Mutation |  | 2 (2%) | 3 (13%) |  |
| WT |  | 118 (98%) | 21 (88%) |  |
| EPHB1 | 4 |  |  | 0.014757 |
| Mutation |  | 1 (1%) | 3 (13%) |  |
| WT |  | 119 (99%) | 21 (88%) |  |
| HNF1A | 3 |  |  | 0.004153 |
| Mutation |  | 0 (0%) | 3 (13%) |  |
| WT |  | 120 (100%) | 21 (99%) |  |
| EZH1 | 2 |  |  | 0.026807 |
| Mutation |  | 0 (0%) | 2 (8%) |  |
| WT |  | 120 (100%) | 22 (92%) |  |
| CD1D | 2 |  |  | 0.026807 |
| Mutation |  | 0 (0%) | 2 (8%) |  |
| WT |  | 120 (100%) | 22 (92%) |  |
